# No Morphological Markers, No Problem: ERP Study Reveals Semantic Contribution to Distinct Neural Substrates Between Noun and Verb Processing in Online Sentence Comprehension

**DOI:** 10.3389/fnins.2019.00957

**Published:** 2019-09-10

**Authors:** Jun Feng, Tao Gong, Lan Shuai, Yicheng Wu

**Affiliations:** ^1^Institutes of Psychological Sciences, Hangzhou Normal University, Hangzhou, China; ^2^Center for Cognition and Brain Disorders, Hangzhou Normal University, Hangzhou, China; ^3^Zhejiang Key Laboratory for Research in Assessment of Cognitive Impairments, Hangzhou, China; ^4^Center for Linguistics and Applied Linguistics, Guangdong University of Foreign Studies, Guangzhou, China; ^5^Educational Testing Service, Princeton, NJ, United States; ^6^Department of Linguistics and Translation, School of International Studies, Zhejiang University, Hangzhou, China

**Keywords:** event-related potential, sentence comprehension, neural processing of nouns and verbs, noun-verb-ambiguous-words, Chinese

## Abstract

Neural mechanisms behind noun and verb processing during the course of language comprehension are ubiquitously separate, yet it remains highly controversial as to which factor, syntax or semantics, should be responsible for this separation. This paper conducted an event-related potential (ERP), sentence comprehension experiment as an attempt to resolve this issue. The experiment used Chinese sentences in the configuration of noun phrase + 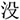 (“not/no”) + noun/verb/noun-verb-ambiguous-word, which excluded grammatical or syntactic factors that could hint at the lexical categories of sentence-final target words. Results showed significantly distinct ERP components of P200, N400, and P600 between noun and verb processing in native speakers, indicating that semantic factors are essential for the differentiated neural mechanisms behind noun and verb processing. Distinct P200, N400, and P600 also manifested between noun and noun-verb-ambiguous-word processing, but not between verb and noun-verb-ambiguous-word processing. This suggests that lacking clues on lexical category renders the dynamic properties of the ambiguous words more salient than the static properties, thus causing interpretation of such words more likely as verbs. This further elaborates the crucial role of semantic factors in noun and verb processing.

## Introduction

Noun and verb processing during sentence comprehension serves as the basis for advanced cognition, and neural mechanisms behind such processing have long been a principal focus of psycholinguists and cognitive neuroscientists (Federmeier et al., 2000; Pulvermüller et al., 2005; Yu et al., 2011). A series of neuroimaging studies, especially functional Magnetic Resonance Imaging (fMRI) and electroencephalography (EEG) ones (see Moseley and Pulvermüller, 2014; Xia et al., 2016 for reviews), have reached a uniform conclusion that nouns and verbs induce differentiated neural mechanisms. For example, verbs are often processed in the left inferior frontal and/or middle temporal cortex, whereas noun processing usually involves temporal and/or parietal areas in the left hemisphere (Damasio and Tranel, 1993; Cappelletti et al., 2008; Gerfo et al., 2008). Research on the human mirror neuron system also reveals that verb processing tends to recruit somatic motor cortex, whereas noun processing does not (Gallese and Lakoff, 2005; Boulenger et al., 2006; Binder and Desai, 2011; Desai et al., 2010, 2011). In addition, it has been repetitively evident that nouns and verbs evoke different event-related potential (ERP) components including P200, N400 and P600 (Preissl et al., 1995; Lee and Federmeier, 2006; Barber et al., 2010).

Despite these findings, there has been of great controversy as to which factor, syntax or semantics, leads to the observed noun-verb distinction (Moseley and Pulvermüller, 2014). For example, studies using artificial words and/or morphologically altered words as stimuli (Tyler et al., 2004; Shapiro et al., 2005 *inter alia*) show that syntactic factors induce distinct neural mechanisms. By contrast, other studies advocate that semantic factors are essential for the neural distinction between noun and verb processing (Lee and Federmeier, 2006; Moseley and Pulvermüller, 2014). For example, the noun-verb neural distinction is still evident in native speakers of a language not relying much on morphological markers to distinguish lexical categories (e.g., Chinese) (Yu et al., 2011; Xia et al., 2016).

Albeit studies using artificial words as stimuli demonstrate that syntactic factors lead to the neural distinction between artificial “noun” and “verb” understanding, processing artificial language is not equivalent to processing natural language, since artificial words do not have concrete meanings, so participants simply pay attention to checking the spellings of target words rather than understanding them. Therefore, these studies are insufficient to explain the distinct neural substrates between noun and verb processing in real communications.

Although studies using Chinese stimuli tend to ascribe the distinct neural mechanisms to semantic factors (Liu et al., 2007, 2008; Yu et al., 2011; Xia et al., 2016), the reason behind this deserves reconsideration. On the one hand, albeit poor in morphological inflections, Chinese uses priming words to denote target words’ part-of-speech. For example, as in Liu et al. (2008) and Xia et al. (2016), 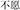 indicates a verb afterward (e.g., 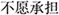, “unwilling to take”), and 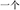 a noun afterward (e.g., 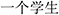, “one student”). In this paradigm, since it has been already primed strongly to expect target words’ part-of-speech before their appearance, participants tend to neglect the meanings of those target words. On the other hand, employing either single words (Zhang et al., 2003; Yu et al., 2011), two monosyllabic words unable to form a meaningful phrase (Liu et al., 2007), or simple noun and modal verb phrases as stimuli (Liu et al., 2008; Xia et al., 2016), none of those studies using Chinese paid sufficient attention to sentence. Many characteristics of Chinese can only be fully expressed at the sentence level (Lv, 1980). First, unlike inflectional languages, words in Chinese seldom change their morphological forms to realize grammatical functions and there exists no one-to-one correspondence between part-of-speech and sentence elements. Second, a huge amount of commonly used verbs in Chinese are noun-verb-ambiguous-words [about 20%, according to Chen (1986), Wu and Luo (1994), and Hu (1995)], such as 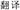 (“to translate”/“translation”/“translator”) or 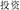 (“to invest”/“investment”). Therefore, it would be hardly possible to identify target part-of-speech without integrating each word’s meaning in the whole sentence. Some neurolinguistics studies recruited such ambiguous words (Li et al., 2004), but did not address the neural mechanisms behind them during sentence comprehension or in a situation lacking sufficient syntactic or contextual clues. Such ambiguous words are highly relevant to exploring neural substrates between noun and verb processing in Chinese understanding, and should be investigated as target words in sentences, together with other sentences having target nouns and verbs.

Our study aims to investigate whether semantic differences between Chinese nouns and verbs contribute to the distinct neural substrates using sentences having a construction of noun phrase (NP) + 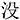
(“no”) + noun/verb/noun-verb-ambiguous-word. There are two reasons for choosing this construction. First, it excludes grammatical or syntactic markers that suggest target word’s part-of-speech. This is because in Chinese, 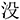, as one of the two mostly used negation words (the other is 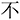), can appear before either nouns or verbs without priming their part-of-speech. For example, native speakers cannot judge the part-of-speech of the target words 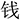 and 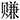 through 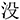 in 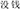 (no money) and 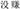 (no earn). Without morphological forms to indicate the lexical categories of these words, the only way to clarify 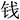 as a noun and 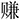 as a verb is via their meanings. Second, NPs commonly appear before either verbs or nouns; and we are unaware of any research reporting that commonly used Chinese NPs could indicate the part-of-speech of the following words. In a visually presented task in which participants are asked to judge the correctness of sentence meaning, they cannot predict a target word’s part-of-speech solely through NP + 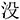. Therefore, with syntactic factors being inherently excluded in such construction, clarifying whether a target word is a verb or a noun is only via semantic factors. Then, based on sentences having such construction, any distinct neural substrates between nouns and verbs processing can reliably reflect the essential role of semantic factors in noun-verb neural separation.

As reported in previous studies (Preissl et al., 1995; Lee and Federmeier, 2006; Xia et al., 2016 *inter alia*), neural separation between noun and verb processing occurs in different stages, and can be reflected by different ERP components such as P200, N400, and P600. P200 represents the initial identification of part-of-speech, and verb processing usually induces significantly larger amplitude than noun processing during the P200 period (Preissl et al., 1995; Liu et al., 2007). N400 denotes clarification of the relationship between predicate and its arguments, and larger amplitude for verb processing than noun processing has been shown in N400 (Federmeier et al., 2000; Lee and Federmeier, 2006). P600 reflects the top–down integration of the meaning of target word and those of other sentence elements, and previous studies have repeatedly reported larger P600 amplitude for verb processing than noun processing (Liu et al., 2008; Xia et al., 2016). In line with these studies, we adopted a similar region-of-interest (ROI) design, and expected and compared P200, N400, and P600 components.

We selected the frontocentral sites as the ROI of P200 and the centroparietal sites as the ROI of N400, as in previous studies. Note that although P600 is commonly expected and reported in the centroparietal area (e.g., Coulson et al., 1998; Federmeier et al., 2000), a recent ERP study (Xia et al., 2016) reported distinct P600 in the frontocentral area between Chinese noun and verb processing. In addition, instead of a unitary phenomenon, P600 represents two aspects with different neural structures (Friederici et al., 2002): the often-found centroparietal-originated P600 is related to syntactic repair, and typically reflects syntactic violation during sentence comprehension, whereas the frontocentral-originated P600 typically reflects syntactic complexity. In our study, the first type of P600 is not expected, since we did not recruit syntactically improbable sentences. In our sentence stimuli, however, the part-of-speech of target words can only be clarified by integrating the meaning of target words with other parts of the sentences, which is highly related to the complexity aspect of P600. Noting these, we selected the frontocentral area as the ROI of P600.

Our experiment showed that Chinese nouns and verbs evoked distinct P200, N400, and P600, suggesting that compared to syntax, semantics plays a more essential role in the observed distinct neural substrates between noun and verb processing. Similar neural differences also manifested between noun and noun-verb-ambiguous-word processing, indicating that in a context lacking sufficient hints at target words’ lexical categories, participants tended to interpret those ambiguous words as verbs rather than nouns, because the dynamic attributes of such words are supposed to be more explicit than the static ones under this context. This further demonstrates the semantic contribution to the separate neural substrates between noun and verb processing.

## Materials and Methods

The experimental protocol of this study was approved by the College Research Ethics Committee of Zhejiang University. The methods were carried out in accordance with the approved guidelines from the College Research Ethics Committee. Informed consents were obtained from all participants.

### Participants

Thirty (15 males, age between 19 and 26, mean = 21, *SD* = 2.5) native Mandarin Chinese speakers participated in the experiment. All were undergraduate or graduate students from Zhejiang University, strongly right-handed as tested by the handedness inventory (Oldfield, 1971), and had normal or corrected-to-normal visions and no history of neurological diseases. None of them majored in linguistics, psychology, or related disciplines at the time of experiment. They voluntarily participated in the experiment, and were paid a proper remuneration after completing it.

### Materials

Our experiment used complete sentences as stimuli. Considering that native speakers could only distinguish target part-of-speech (noun or verb) after reading the whole sentence and understanding target word’s meaning in the sentence, we placed target word at the end of each sentence and excluded possible hints on target part-of-speech.

Using sentence-final words as critical (target) words is a common practice in ERP experiments. Among them, those involving syntactic violations report a “sentence-ending global effect,” i.e., participants keep considering the syntactic structure of the whole sentence even after the appearance of the final word, which evokes more complex ERP components than classic language processing, such as N400-like or N700 components (Holcomb et al., 1999; West and Holcomb, 2000; Hagoort, 2003). By contrast, others using sentences involving semantic issues (e.g., context relatedness or word congruency) seldom report such effect or ERP components (Federmeier and Kutas, 1999; Hagoort and Brown, 2000; Federmeier et al., 2002; Borovsky et al., 2010). Since the materials in our study contained semantic issues only and syntactic issues were screened out, using sentence-final words as critical (target) words would not cause potential problems.

The stimulus sentences in our study had a special construction: noun phrase (NP) + 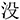 + target word (noun/verb/noun-verb-ambiguous-word). 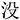 and 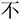 are two most commonly used negation words in modern Chinese (Lv, 1980; Zhu, 1985). One difference between them is that 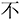 is subjective, e.g., 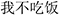 [I don’t (want to) eat], while 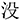 simply gives an objective negation, e.g., 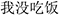 (I did not eat) (Hou, 2016). In addition, 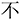 is rarely used to negate nouns, unless they have certain properties, e.g., 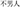 (not man) (Lv, 1980, p. 363; Ma, 2004, p. 108). By contrast, 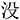 is freely used before nouns or verbs in a sentence, without revealing their lexical categories. For example, 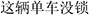 is a semantically ambiguous sentence containing a noun-verb-ambiguous-word 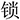 meaning either “locker” or “to lock.” Accordingly, the sentence could be interpreted as either “This bike has no lockers” or “This bike is not locked.” Considering that 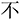 and 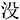 are the only negation words in Chinese and 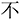 rarely negates nouns, in principle, there should be no preference for 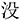 to negate nouns or verbs (otherwise, Chinese cannot fulfill a complete function of negation, and we are not aware of any research reporting such preference).

Along with previous studies using Chinese nouns and verbs, we adopted three types of monosyllabic or disyllabic target words: nouns, e.g., 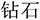 (“diamond”); verbs, e.g., 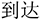 (“to arrive”); and noun-verb-ambiguous-words, e.g., “
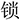
” (“locker”/“to lock”). In principle, it would be ideal to control the number of syllables in target words, as in some previous lexical/phrase comprehension experiments (e.g., Li et al., 2004). In practice, there are more disyllabic noun-verb-ambiguous-words than monosyllabic ones, and the ERP framework of sentence comprehension does not strictly require that target words have equal numbers of syllables. In addition, according to the Modern Chinese Dictionary (The Commercial Press, 2012), the target nouns and verbs can never be used as other lexical categories, and the noun-verb ambiguous-words can be used only as nouns or verbs but no other categories. Furthermore, all the target words have relatively high frequencies according to the corpus by the Ministry of Education of the People’s Republic of China^1^. This corpus contains over 150,000 Chinese characters (considering the rather free combination of Chinese characters to make words, the number of words in this corpus is incalculable). According to the corpus, the mean log-transformed (base *e*) frequency of the selected nouns in our study is 0.111‰ (*SD* = 0.15‰), that of the selected verbs is 0.111‰ (*SD* = 0.176‰), and that of the selected noun-verb-ambiguous-words is 0.593‰ as verbs (*SD* = 0.071‰) and 0.532‰ as nouns (*SD* = 0.179‰). We analyzed these frequencies by one-way ANOVA, and found no significant differences between the selected verbs and nouns [*F*(1,62) = 0.096, *p* = 0.757, η^2^ = 0.002] and between the selected ambiguous words used as verbs and used as nouns [*F*(1,62) = 0.032, *p* = 0.858, η^2^ = 0.001]. This suggests the basically equal ambiguity of the part-of-speech of the ambiguous words. Finally, instead of participating in the ERP experiment, additional 15 participants were recruited to rate the difficulty of the target words’ meanings with five scales: very simple, simple, neutral, hard, and very hard to understand. All the target words were rated as very simple to understand. This indicates that the difficulty of the target words’ meanings would not greatly affect the experimental results.

There were two types of stimulus sentences. The first type was both syntactically and semantically sound, e.g., with a noun as the target word, 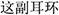 (this pair of earrings) + 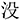 (no) + 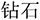 (diamonds) (“This pair of earrings has no diamonds”); with a verb as the target word, 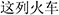 (this train) + 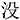 (no) + 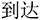 (arrive) (“This train has not arrived”); and with a noun-verb-ambiguous-word as the target word, 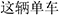 (this bike) + 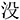
(no) + 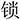 (lock) (“This bike has not been locked”/“This bike has no locker”). Here, “ +” is arbitrarily inserted to illustrate structure components, there were no such delimiter in the real stimuli. The second type was syntactically correct but semantically improbable, e.g., with a noun as the target word, 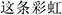 (this rainbow) + 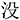 (no) + 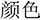 (color) (“This rainbow has no color”); and with a verb as the target word, 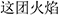 (this flame) + 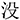 (no) + 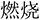 (burn) (“This flame has not burned”).

Note that syntactically improbable but semantically plausible sentences were not employed in our experiment. Since the main focus of our study is to examine whether the semantic factors (i.e., different semantics between nouns and verbs *per se*) alone could lead to distinct neural substrates, we tried avoiding syntactic factors that would be likely to affect sentence comprehension (e.g., we use 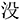 to exclude hints on the target word’s lexical category). Syntactically incorrect but semantically plausible sentences would inevitably influence participants’ sentence understanding. More precisely, they tend to seriously interfere with the investigation of the role played by semantic factors. Additionally, sentences with incorrect syntax and improbable semantics were also excluded. Our study aims to investigate whether distinct neural substrates between noun and verb processing are induced by semantic factors alone. Therefore, the syntactic aspects of the stimulus sentences should remain consistent.

All the sentence stimuli had the same construction of NP + 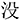 + target word (noun/verb/noun-verb-ambiguous-word). Here, each NP had three or four characters, such as 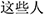 (these people) or 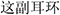 (this pair of earrings). All the stimuli belonged to 6 conditions, each having 32 sentences with distinct target words. Table 1 shows the examples of these sentences (Supplementary Table S1 lists the complete materials). Conditions 1 to 3 contained semantically correct sentences: sentences in condition 1 had noun-verb-ambiguous-words as target words, those in condition 2 had verbs as target words, and those in condition 3 had nouns as target words. Conditions 4 and 5 contained semantically improbable sentences: sentences in condition 4 had verbs as target words, and those in condition 5 had nouns as target words. Noun-verb-ambiguous-words never occurred as target words in semantically improbable sentences. No previous research has ever put such ambiguous words into incongruent sentences as stimuli. A possible reason for not doing so is that the contextual incongruity of the whole sentence would induce global effects much stronger than that at the lexical level (Kotchoubey and El-Khoury, 2014). In addition, the global contextual information shows a slightly delayed effect when it is inconsistent with the local information (Kambe et al., 2001). Therefore, if noun-verb-ambiguous-words appear as sentence-final target words in incongruous sentences, it would be rather difficult to discriminate whether the induced effects are due to sentence incongruity or lexical ambiguity. Finally, condition 6 contained sentences with wrongly matched NP and VP, e.g., 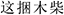 (this bundle of firewood) + 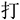 (hit/make) + 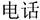 (phone) (“This bundle of firewood makes a phone call”). The purpose of adopting these obviously improbable sentences was to test whether a participant was seriously executing the task. If he/she had a very low accuracy in this condition, his/her data were eliminated from analyses. Considering the occasional or unintentional key pressing errors, we only accepted the data of participants having over 80% accuracy in condition 6.

**TABLE 1:** Examples of sentence stimuli.

	**Condition 1–5**				
**Condition**	**NP**	**Negative word**	**Target word**	**Target category**	**Correctness**
1	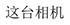 This camera	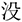 No	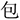 Pack	Ambiguous	Correct
2	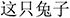 This rabbit	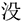 No	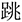 Jump	Verb	Correct
3	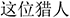 This hunter	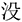 No	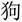 Dog	Noun	Correct
4	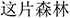 This forest	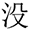 No	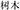 Tree	Noun	Improbable
5	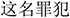 This convict	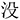 No	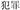 Crime	Verb	Improbable

	**Condition 6**				
**Condition**	**NP**		**VP**		**Correctness**

6	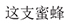		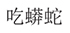		Improbable
	This bee		Eat python		

*Column “Target category” indicates the noun/verb/noun-verb ambiguous category of the target word, and column “Correctness” indicates whether the sentence in each condition is semantically correct or improbable.*

### Procedure

The experiment was carried out in a soundproof and electrically shielded room, where participants sit comfortably in front of a 17-inch CRT screen. The resolution of the screen was 1024 × 768, the distance between the screen and participants’ eyes was approximately 100 cm, and every character in a sentence was displayed in the font of Song, with a size of 60 pixels to ensure that it could be seen clearly. Throughout the experiment, participants were told to try remaining quiet and still.

Prior to the formal experiment, there were practice trials, in which participants were asked to judge whether each sentence appearing on the screen was logically correct or not, by pressing the corresponding keyboard buttons as accurately as possible. The stimuli used in the practice trials were different from those in the formal experiment, and participants moved to the formal experiment till achieving over 90% accumulated correctness in the practice trials.

**FIGURE 1:**
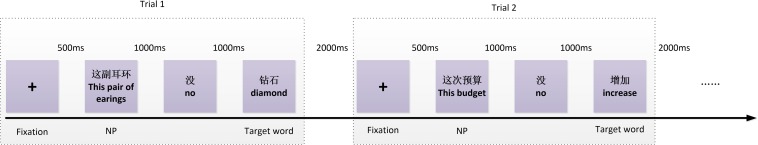
Trial procedure. Each trial contains one sentence.

In the formal experiment, the total 192 sentences in conditions 1 to 6 were divided into 16 blocks, each containing 12 sentences, 2 from each condition. Each sentence was shown in one trial. In each block, there were 2 trials for each of the six conditions. The procedure of each trial was shown in Figure 1. In each trial, a white crossing first appeared for 500 ms at the center of the screen to catch participants’ attention. Then, the stimulus sentence was shown in the center of the screen in the sequence of NP, 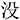, and target word, e.g., 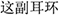, 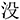, and 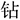

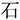
, duration of each was 1000 ms. Participants were asked to judge whether each sentence was logically correct or improbable by pressing the corresponding keyboard buttons within 2000 ms. When a judgment was made within 2000 ms, the next trial started automatically. If no judgment was made within 2000 ms, this trial was considered incorrectly judged, and the next trail started automatically. Trials appeared randomly in a block. Participants were allowed to take 1-min inter-block break. The whole experiment lasted about half an hour.

### EEG Recording and Preprocessing

We used a Neuroscan Synamps2 system to record the EEG data, and the Scan 4.5 software provided by NeuroScan, Inc. to analyze the data. During the experiment, participants wore a Quick-Cap 64 elastic cap for data recording. We located one pair of electrodes above and below the left eye of a participant for the VEOG signal and another pair outside of outer canthi of both eyes for HEOG. All recording used bilateral mastoids as the reference point and the EEG data were referenced online. The impedance of each electrode was kept below 5 k Ω. The band pass filtering was set between 0.05 and 100 Hz, and the sample frequency was set to 1000 Hz. Ocular movements and other artifacts were excluded from further analysis according to the threshold ±100 μV, and the ERP data were computed over a range of 200 ms before and 800 ms after the onset of the target words. There were fewer than 9% rejected trials in each of conditions 1 to 5 (in each condition, there were data of 960 trials recorded (32 trials times 30 participants), 38 trials in condition 1, 52 in condition 2, 40 in condition 3, 84 in condition 4, and 69 in condition 5 were rejected during artifact cleaning).

**FIGURE 2:**
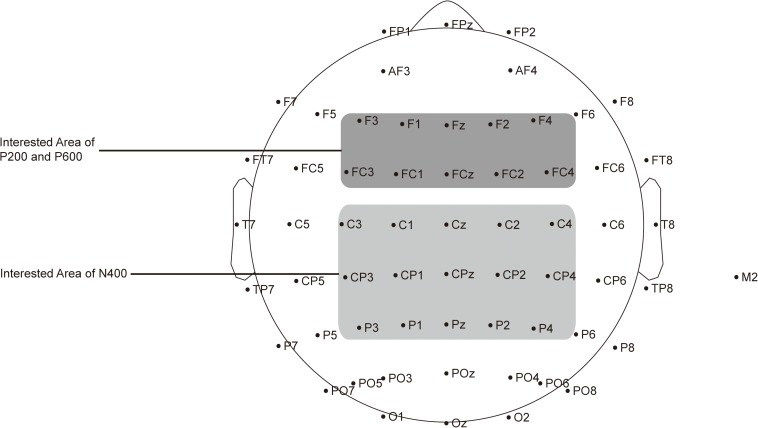
Distribution of the 64 electrodes over the scalp used to record EEG signals. Electrodes used to capture P200 and P600 are marked in dark gray, and those used to capture N400 are marked in light gray.

Data for analysis included the behavioral and ERP data, which are both recorded online. The behavioral data were participants’ judgment accuracies (ACC) and reaction times (RT) for each sentence. The ERP data were recorded using different ROIs. In line with early studies, we focused on three ERP components closely relevant for language processing: P200 (its time range was set to 100–300 ms) (Zhang et al., 2003; Liu et al., 2007), N400 (300–500 ms) (Kutas and Federmeier, 2000; Xia et al., 2016) and P600 (500–800 ms) (Friederici et al., 2002; Liu et al., 2008). As shown in Figure 2, the signals from the electrodes Fz, FCz, F3, F4, F1, F2, FC3, FC4, FC1, and FC2 were used to calculate the average amplitude of P200 and P600 (Friederici et al., 2002; Xia et al., 2016), and those from Cz, CPz, Pz, C3, C4, C1, C2, CP3, CP4, CP1, CP2, P3, P4, P1, and P2 were used to calculate the average amplitude of N400 (Kutas and Federmeier, 2000; Liu et al., 2007). As argued in the Introduction, we selected the frontocentral area as the ROI to capture the complexity-related P600 (Friederici et al., 2002; Xia et al., 2016).

### Data Analysis

For each type of data (ACC, RT, and the three ERP components), we conducted a two-way repeated measures ANOVA with Greenhouse–Geisser correction to analyze the effects of lexical category (2 levels: verbs and nouns), semantic correctness of sentence (2 levels: correct and improbable), and the interaction between the two. If significant main effect(s) or interaction was reported, separate pair-wised *t*-tests were applied to detect significant differences respectively between verbs in semantically correct and improbable sentences, between nouns in semantically correct and improbable sentences, between verbs and nouns in semantically correct sentences, and between verbs and nouns in semantically improbable sentences.

We also conducted a one-way repeated measures ANOVA with Greenhouse–Geisser correction on the effect of lexical category (3 levels: noun-verb-ambiguous-words, verbs, and nouns) in semantically correct sentences. If a significant effect was reported, separate pair-wised *t*-tests were applied to detect significant differences respectively between verbs and nouns, between noun-verb-ambiguous-words and verbs, and between noun-verb-ambiguous-words and nouns.

In the ANOVA tests, we controlled the family wise Type I error probability by setting the critical *p-*value for identifying significant effects as.05/10 = 0.005 [10 was set according to 5 type of data (ACC, RT, P200, N400, and P600) times 2 ANOVA tests (one-way repeated measure and two-way repeated measure)]. The critical *p*-value for the separate pair-wised *t*-tests after the two-way repeated measures ANOVA was set to.05/4 ≈0.013 (4 was the number of comparisons), and the critical *p*-value for the separate pair-wised *t*-test after the one-way repeated measures ANOVA was set to.05/3 ≈0.017 (3 was the number of comparisons). All the tests were executed in SPSS 22 (Arthur et al., 2013; Barry, 2013).

## Results

The judgment accuracies of all 30 participants in the sentences from condition 6 were over 90% (mean = 0.974, *SD* = 0.030), indicating that all of them executed the task seriously.

### Behavioral Data

The mean RTs of the target nouns, verbs, and noun-verb-ambiguous-words in the sentences from conditions 1, 2, and 3 were 819.08, 762.73, and 765.97 ms, respectively, and the mean accuracies (ACCs) were 0.740, 0.860, and 0.800, respectively.

For ACCs, the two-way repeated measures ANOVA revealed that: lexical category had a significant main effect [*F*(1,29) = 19.356, *p* < 0.001, η^2^ = 0.4], but semantic correctness did not (*p* = 0.121) and the two interacted insignificantly (*p* = 0.119). Separate pair-wised *t*-tests analyses showed that sentences with target verbs had significantly higher ACCs than those with target nouns in the semantically correct sentences (*p* < 0.013), and also in the semantically improbable sentences (*p* < 0.013). The one-way repeated measures ANOVA also revealed a significant main effect of lexical category in the semantically correct sentences [*F*(1,29) = 7.96, *p* < 0.005, η^2^ = 0.215]. Separate pair-wised *t*-tests analyses showed that: the ACCs of sentences having target verbs were significantly higher than those having target nouns (*p* < 0.017), and the ACCs of sentences having target noun-verb-ambiguous-words were also significantly higher than those having target nouns (*p* < 0.017); but the ACCs between sentences having target noun-verb-ambiguous-words and those having target verbs were not significantly different (*p* = 0.7471). Detailed statistics are in Supplementary Table S2.

For RTs, the two-way repeated measures ANOVA showed no significant effects of lexical category (*p* = 0.156) or semantic correctness (*p* = 0.165), nor interaction between the two (*p* = 0.536). The one-way repeated measures ANOVA also showed no significant effect of lexical category (*p* = 0.132). Detailed statistics are in Supplementary Table S3.

### ERP Data

#### P200

During the 100–300 ms time window, the two-way repeated measures ANOVA showed that: lexical category had a significant main effect on the amplitude of P200 [*F*(1,29) = 67.159, *p* < 0.001, η^2^ = 0.698], but semantic correctness did not (*p* = 0.24), and there were no significant interactions between the two (*p* = 0.648). Separate pair-wised *t*-tests analyses showed that verbs had significantly bigger P200 amplitudes than nouns in the semantically correct (*p* < 0.001) and improbable (*p* < 0.001) sentences. The one-way repeated measures ANOVA also revealed a significant main effect of lexical category [*F*(1,29) = 14.063, *p* < 0.001, η^2^ = 0.327]. Separate pair-wised *t*-tests analyses showed that: the amplitudes of P200 evoked by verbs were significantly bigger than those evoked by nouns (*p* < 0.001), the amplitudes of P200 evoked by noun-verb-ambiguous-words were also significantly bigger than those evoked by nouns (*p* < 0.001), but no significant difference was found in P200 between verbs and noun-verb-ambiguous-words (*p* = 0.820). Detailed statistics are in Supplementary Table S4.

**FIGURE 3:**
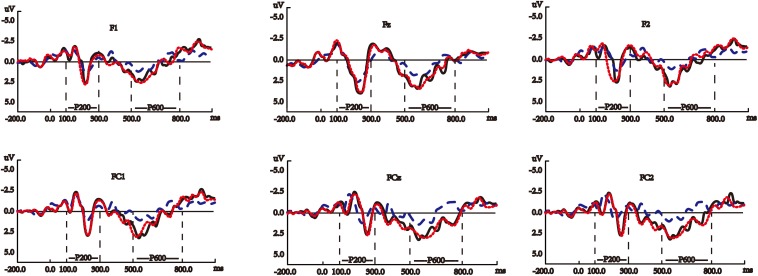
Average waves elicited by the target nouns (blue dashed lines), verbs (black solid lines), and noun-verb ambiguous words (red dotted lines) on P200 (within left two vertical lines) and P600 (within the right two vertical lines) in the semantically correct sentences. The *y*-axes in these panels are negative up. All the panels show the waveforms at the six electrodes for P200 and P600, the waveforms at the other electrodes are shown in Supplementary Figure S1.

**FIGURE 4:**
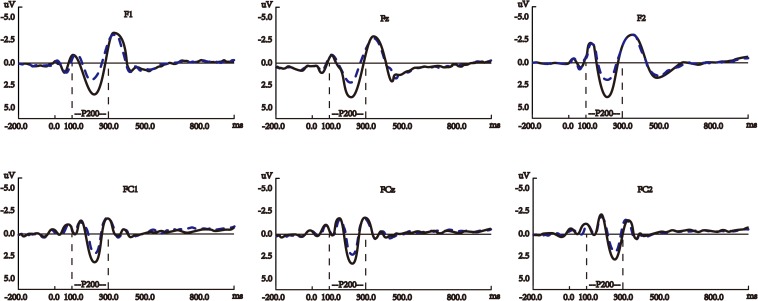
Average waves elicited by the target nouns (blue dashed lines) and verbs (black solid lines) on P200 in the semantically improbable sentences. The *y*-axes in these panels are negative up. All the panels show the waveforms at the six electrodes for P200, the waveforms at the other electrodes are shown in Supplementary Figure S2.

Figure 3 shows the average waves elicited by the target nouns, verbs, and noun-verb-ambiguous-words on P200 in the semantically correct sentences (see Supplementary Figure S1 for the waveforms of the other electrodes for this ERP component). Figure 4 shows the average waves elicited by the target nouns and verbs on P200 in the semantically improbable sentences (see Supplementary Figure S2 for the waveforms of the other electrodes for this ERP component).

#### N400

During the 300–500 ms time window, the two-way repeated measures ANOVA showed that: lexical category had a significant main effect on the amplitude of N400 [*F*(1,29) = 23.445, *p* < 0.001, η^2^ = 0.447], so did semantic correctness [*F*(1,29) = 185.976, *p* < 0.001, η^2^ = 0.865], but there were no significant interactions between the two (*p* = 0.025). Separate pair-wised *t*-tests analyses showed that verbs had significantly bigger N400 amplitudes (note that N400 is a negative component) than nouns in the semantically correct sentences (*p* < 0.001), but not in the semantically improbable sentences (*p* = 0.536), and nouns (*p* < 0.001) and verbs (*p* < 0.001) in the semantically improbable sentences evoked significantly bigger N400 amplitudes than those in the semantically correct sentences. The one-way repeated measures ANOVA also revealed a significant main effect of lexical category [*F*(1,29) = 13.318, *p* < 0.001, η^2^ = 0.315]. Separate pair-wised *t*-tests analyses showed that: the amplitudes of N400 evoked by verbs were significantly bigger than those evoked by nouns (*p* < 0.001), the amplitudes of N400 evoked by noun-verb-ambiguous-words were also significantly bigger than those evoked by nouns (*p* < 0.001), but no significant difference was found in N400 between verbs and noun-verb-ambiguous-words (*p* = 0.969). Detailed statistics are in Supplementary Table S5.

**FIGURE 5:**
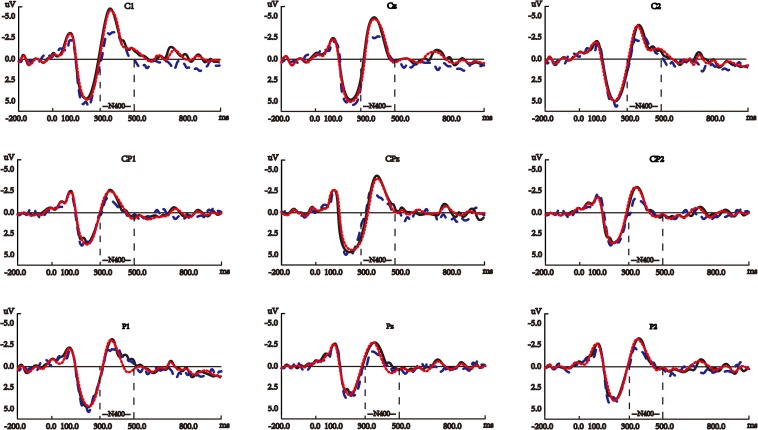
Average waves elicited by the target nouns (blue dashed lines), verbs (black solid lines), and noun-verb ambiguous words (red dotted lines) on N400 in the semantically correct sentences. The *y*-axes in these panels are negative up. All the panels show the waveforms at the nine electrodes for N400, the waveforms at the other electrodes are shown in Supplementary Figure S3.

Figure 5 shows the average waves elicited by the target nouns, verbs, and noun-verb-ambiguous-words on N400 in the semantically correct sentences (see Supplementary Figure S3 for the waveforms of the other electrodes for this ERP component).

#### P600

During the 500–800 ms time window, the two-way repeated measures ANOVA showed that: lexical category had a significant main effect on P600 [*F*(1,29) = 9.412, *p* = 0.005, η^2^ = 0.245], so did semantic correctness [*F*(1,29) = 189.503, *p* < 0.001, η^2^ = 0.867], but there were no significant interactions between the two (*p* = 0.286). Separate pair-wised *t*-tests analyses showed that verbs had significantly bigger P600 amplitudes than nouns in the semantically correct sentences (*p* < 0.001) but not in the semantically improbable sentences (*p* = 0.374), and nouns (*p* < 0.001) and verbs (*p* < 0.001) in the semantically improbable sentences evoked significantly bigger P600 amplitudes than those in the semantically correct sentences. The one-way repeated measures ANOVA also revealed a significant main effect of lexical category [*F*(1,29) = 21.016, *p* < 0.001, η^2^ = 0.42]. Separate pair-wised *t*-tests analyses showed that: the amplitudes of P600 evoked by verbs were significantly bigger than those evoked by nouns (*p* < 0.001), the amplitudes of P600 evoked by noun-verb-ambiguous-words were also significantly bigger than those evoked by nouns (*p* < 0.001), but no significant difference was found in P600 between verbs and noun-verb-ambiguous-words (*p* = 0.716). Detailed statistics are in Supplementary Table S6.

Figure 3 also shows the average waves elicited by the target nouns, verbs, and noun-verb-ambiguous-words on P600 in the semantically correct sentences (see Supplementary Figure S1 for the waveforms of the other electrodes for this ERP component).

Apart from the waveforms, Supplementary Figure S4 shows the topographies of the ERP components evoked by the target nouns, verbs, and noun-verb-ambiguous-words in the semantically correct sentences. Comparisons among Supplementary Tables S4–S6 show that nouns and verbs only evoked significantly different P200 component in the semantically improbable sentences. Accordingly, Supplementary Figure S5 shows the topographies of P200 evoked by the target nouns and verbs in the semantically improbable sentences. All the topographies had frontocentral (P200 and P600) or centralparietal (N400) distributions, without obvious left or right lateralization. These are consistent with previous findings about these components in lexical, phrasal, and sentence comprehension (Preissl et al., 1995; Kellenbach et al., 2002; Friederici, 2011; Kutas and Federmeier, 2011).

## Discussion

Our study showed that verbs evoked significantly larger P200, N400, and P600 than nouns in sentences without part-of-speech markers and priming effects. Similar differences also existed between noun-verb-ambiguous-words and nouns, indicating that without explicit clues on lexical categories of noun-verb-ambiguous-words, the verbal senses of these words became more salient than their nominal senses, thus prompting the participants to interpret these words as verbs. All these indicate that semantic factors are essential for the separate neural mechanisms between noun and verb processing.

Distinct neural mechanisms between noun and verb processing shown in our study are in line with the universal properties of human languages. Although not all languages have explicit notions of nouns and verbs, such as Tongan and Cayuga (Broschart, 1997; Kaufman, 2009), every language has its own way of expressing and distinguishing actions (“becoming”) and entities (“permanence”) (Langacker, 1987; Croft, 2000; Baker, 2003). Nouns and verbs differ inherently in major linguistic aspects (Laudanna and Voghera, 2002). Nouns are often used to represent lifeless objects or living entities, thus being relatively more stable and less affected by time change, whereas verbs often describe actions or states, thus being relatively more dynamic and sensitive to time change (Frawley, 2013). Nouns can be used not only as the subjects or objects of verbs, but also as the objects of prepositions, while verbs usually serve as predicates, indicating how subjects and objects instigate and are affected by the actions expressed by verbs. Moreover, these two lexical categories display a significant difference in terms of grammatical markers, particularly in inflectional languages: the part-of-speech marker of a noun often indicates gender, number (countable/uncountable), or case of permanence that it represents, while the morphological marker of a verb normally shows time, aspect, and tense of the action denoted by it. Last but not least, at the pragmatic or discourse level, nouns are typically used to express themes, whereas verbs are employed to discuss themes. As is evident in our and other studies, many of the differences between nouns and verbs are caused by their inherently different semantics, upon which syntactic factors like inflection are built. This echoes the “semantics driving syntax” hypothesis on language evolution (Schoenemann, 1999).

As an isolating language, Chinese is well-known to be poor in morphological markers, and many of its grammatical meanings are realized at the sentence level. Therefore, using Chinese as stimuli bears unique advantages in investigating the essential cause of the distinct neural mechanism between noun and verb processing. In addition, compared to previous research also using Chinese as stimuli, our study focuses on noun and verb processing during sentence comprehension, which is more consistent with natural language understanding in reality. Furthermore, the experimental stimuli used in our study are carefully designed to largely exclude syntactic factors, which helps reveal and investigate the key roles of semantics in the observed separate neural processing between nouns and verbs.

In the following sections, we discuss the possible reasons why the ERP components evoked by nouns and verbs differ in amplitudes and the particular characteristics of the noun-verb-ambiguous-words.

### Differences in the ERP Components Evoked by Nouns and Verbs

#### P200

P200 is an early component during language processing. Having a short duration, it is generally considered to reflect the initial stage of comprehension, i.e., the initial recognition of lexical information (Friederici et al., 1999; Martin et al., 2006; Wu et al., 2012), and this process is highly automated (Holcomb et al., 1992; Martín-Loeches et al., 1999; Barber et al., 2004; Regel et al., 2011).

In our study, significant differences were found between verbs and nouns in either semantically correct or improbable sentences (see Supplementary Table S4) and Figures 3, 4 collectively reveal that at the time window of P200, verb processing started to show differences from noun processing by eliciting larger (in amplitude) P200, regardless of semantic correctness of sentence. This suggests that during the P200 period the language processing system is already sensitive to the categorical distinction between verbs and nouns, which is consistent with early findings (Preissl et al., 1995; Federmeier et al., 2000; Liu et al., 2007). In addition, among the three ERP components, nouns and verbs only evoked significantly different P200 in the semantically improbable sentences. This indicates that at the P200 time window the language processing system has not received sufficient information and cognitive resources to judge sentence correctness, which is in line with early study reporting no association between P200 and sentence correctness (Liu et al., 2011).

Although some studies described P200 as a marker of the level of expectancy for a particular item (e.g., Viswanathan and Jansen, 2010), many studies on noun and verb processing in visually presented tasks seldom considered P200 as an index of the expectation of a specific target word category, no matter whether the experimental tasks included priming words or not. For example, Preissl et al. (1995) asked participants to perform a lexical decision task in German and revealed significantly distinct P200 between verb and noun processing. As a task including no priming effects, there is no reason for participants to expect that every target word that appears should be a verb or a noun. In addition, in Liu et al. (2007) and Xia et al. (2016), each target word was presented after a priming word, which could indicate the lexical category of the target word [e.g., 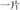 (one piece of) indicates that 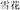 (snowflake) is a noun, and 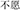 (unwilling to) indicates that 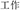 (work) is a verb]. The priming words provided the same strong expectations for the lexical categories of target words, yet verbs were found to still evoke significantly larger P200. These findings suggest that the relationship between P200 and expectancy is reasonably minor for part-of-speech understanding.

In our view, the difference in P200 between verbs and nouns can be ascribed to the fact that the lexical information of the two lexical categories is inherently different, and such difference can be identified automatically at the early stage of lexical processing. Nouns usually represent objects or concepts, and are in a quiescent state; by contrast, verbs often express actions and relationship between objects and subjects. These explicit lexical features serve as the base for later recognition and understanding.

#### N400

Verbs evoked significantly larger N400 than nouns in the semantically correct sentences (see Figure 5), and part-of-speech showed significant main effects in both one-way and two-way repeated measures ANOVA (see Supplementary Table S5). Our results also demonstrated that during the N400 time window, compatible with the classic N400 studies (Hagoort et al., 2004; Kutas and Federmeier, 2011), the language processing system is sensitive to semantic correctness (semantic correctness showed a significant main effect much bigger than that of part-of-speech in the two-way repeated measures ANOVA, and significant difference existed respectively between verbs in the semantically correct and improbable sentences and between nouns in the semantically correct and improbable sentences, as shown in the separate pair-wised *t*-tests in Supplementary Table S5). Moreover, it is worth noting that N400 evoked by verbs and nouns in the semantically improbable sentences showed no significant difference (see Supplementary Table S5). In these sentences, the part-of-speech of target words was correct, but their semantics mismatched the other parts of the sentences, which rendered the part-of-speech of target words to be unessential at the N400 phase of sentence comprehension, thus resulting in no significant difference between verbs and nouns in these sentences.

The N400 effect has been remarked particularly as an index of lexical and semantic processing. Recent studies have provided clear evidence that larger N400 amplitude represents both the top-down and bottom-up aspects of language processing (see Lotze et al., 2011 for a review). In the top–down processing, larger N400 amplitude is induced by the target word’s violation with contextually induced lexical preactivation, in which case many crucial clues for full understanding need to be abstracted within context rather than target word *per se* (Lau et al., 2008). In the bottom–up processing (there also exist a wide variety of influencing factors, see Hagoort and van Berkum, 2007), larger N400 amplitude not only represents an informative mismatch between the target word’s semantics and its information status (Van Berkum et al., 2008) (e.g., a kid’s voice saying *Every evening I drink some wine before I go to sleep*), but also reflects the difficulties in integrating the target word’s sense with other parts of the sentence, which is a within-sentence procedure compared to the contextually top–down processing. In our study, each sentence was complete and independent, which did not require the top–down procedure for contextual information, so the issue about target word’s information status was not involved. However, the bottom–up within-sentence integration was still needed, as indicated by the observed N400 effect.

Compared to P200, which represents the initial lexical recognition, the N400 phase is more complicated and in-depth, in particular, nouns as verbs’ thematic roles (who does what to whom) are assigned to verbs in this phase (Friederici, 2011); that is, verbs usually have more complicated semantics than nouns and certain words need to be organized for understanding at this higher level of processing. During the organization of word meanings, a verb’s semantics is usually handled first by counting the number of its arguments. In our study, each target verb in a sentence had only one argument, e.g., 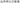 (“This idea has not expanded”). It would be more difficult when a verb takes two or three arguments. In addition, the semantic information accompanying a verb is more than that accompanying a noun. As in the above example, the verb 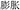 (“to expand”) expresses an action, which concerns the subject of it (“idea”), the place where it occurs (“in somebody’s brain”), the pattern of it (“growing at a very fast pace”), and the consequence of it (“far more than its proper degree, or even explode”). Therefore, processing verbs requires more cognitive resources than processing nouns, thus causing verbs to evoke larger N400 amplitudes.

#### P600

Verbs evoked significantly larger P600 than nouns in semantically correct sentences (see Figure 3), and part-of-speech showed significant main effects in both one-way and two-way repeated measures ANOVA (see Supplementary Table S6). The main effect of part-of-speech indicates that the neural separation between Chinese verbs and nouns continues in this later stage of sentence comprehension. In addition, our results demonstrated that during the P600 time window, the language processing system remains sensitive to semantic correctness (semantic correctness had a significant main effect much larger than that of part-of-speech, and significant difference existed respectively between verbs in the semantically correct and improbable sentences and between nouns in the semantically correct and improbable sentences, as shown in Supplementary Table S6).

Many previous studies have claimed that compared to semantics violation, P600 is much more related to syntax violation (Hagoort et al., 1993; Osterhout and Nicol, 1999; Osterhout et al., 2002). By contrast, many recent studies have revealed that P600 has closer relationship with sentential semantic integration than with reanalysis of syntactic structure (Kolk et al., 2003; Hoeks et al., 2004; Kim and Osterhout, 2005; Kuperberg et al., 2007). This is echoed by our results that P600 was elicited in the sentences with legitimate syntax yet improbable semantics. The semantically improbable sentences used in our experiment had the same feature as the abovementioned sentences, and our results confirmed the sensitivity of P600 to semantics.

Similar to N400, during the time window of P600, verbs and nouns in the semantically improbable sentences showed no significant differences, and part-of-speech and semantic correctness interacted insignificantly (see Supplementary Table S6). In addition, in the semantically improbable sentences, the target words’ categories (syntax) were correct yet their semantics was not, thus making the target words’ categories less essential at the P600 phase of sentence comprehension. Therefore, there was no significant difference in P600 between verbs and nouns in the semantically improbable sentences. Since the only essential factor here was semantic, the interaction between part-of-speech and semantic correctness also remained insignificant.

As has been described earlier, P600 is not a unitary component but indicates two different aspects at the late stage of sentence processing, namely semantic integration and syntactic repair. These two aspects have different neural substrates and distinct distributions (Friederici et al., 2002). The repair-related P600 is usually shown in the centroparietal area (e.g., Coulson et al., 1998; Federmeier et al., 2000), reflecting whether there exist syntax violations. The integration-related P600 is often found in the frontocentral area (Friederici et al., 2002; Xia et al., 2016), reflecting the difficulty of integrating target word’s meaning into the whole sentence, in other words, this type of P600 is integration related. In our study, syntactically violated sentences were excluded on purpose, and the only way to identify the part-of-speech of a target word was to integrate its semantics with other sentential components. Therefore, the repair-related P600 was not expected, but the frontocentral P600 (integration related) was. In line with Xia et al. (2016), in our study, verbs induced significantly larger P600 than nouns in the frontocentral area.

According to Friederici (2011), during sentence comprehension, if the meaning of a sentence is clear, sentential comprehension is concluded at the phase of N400. However, if it is rather difficult to integrate semantic information of a sentence, due to existing ambiguity or lacking contextual clues, an extra phase represented by P600 would be involved for the final and comprehensive integration of semantic relations among all sentential elements, including contextual clues. In addition, for sentences lacking contextual information or having defects, more background knowledge would be taken into account. Per these views, P600 actually represents the highest level of semantic understanding during sentence comprehension.

In our study, verbs were more difficult than nouns in terms of integration with sentential components. Such integration manifested differently in the periods of N400 and P600, respectively as a bottom–up and a top–down process (Friederici, 2011). In the N400, bottom–up process, the whole meaning of a sentence is pieced together by each word. However, in the P600, top–down process, the more precise meaning of a word is restated after the whole sentence is comprehended. This bottom–up and top–down mixed comprehension is not limited to sentence comprehension, but also occurs in comprehension of other language components such as lexical tones in Chinese (Shuai and Gong, 2014). Since the negation word 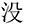 can negate either verbs or nouns or both, participants had to reintegrate, in a top–down manner, to determine whether 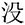 denied the action of the subject or the properties of the subject. Between the two, the negation of action involves more elements, such as the object, result, extent, place, and condition of that action. Therefore, integrating the negation of action requires more semantic information than integrating the negation of subject properties. In addition, it remains controversial as to whether there exists a positive or negative correlation between the amplitude of P600 and the difficulty of sentential integration (Osterhout and Holcomb, 1992; Friederici and Meyer, 2004; Hagen et al., 2006; Liu et al., 2008). The reason why verbs evoked larger P600 in our experiment is that verbs are more difficult than nouns to integrate within the sentential structure. In this sense, our study supports the view that the greater the difficulty of semantic integration, the greater the P600 amplitude.

In sum, the observed P200, N400, and P600 effects all reflect the significantly distinct neural substrates between noun and verb processing, and indicate that the reason behind this phenomenon is the inherent semantic difference between nouns and verbs; to be specific, in our study which excluded syntactic factors, the semantic factors led to the distinct neural substrates between noun and verb processing. The semantic difference between nouns and verbs lies not only in the fact that nouns are often referred to entities and verbs to actions, but also the unequal amounts of semantic elements involved in nouns and verbs. As shown in many studies explaining the inherent difficulties of verbs (e.g., Gentner, 1982; Snedeker and Gleitman, 2004), the number of senses of a verb is much more than an action itself, but its subject (and object), method and path, duration, outcome, and so on. Verbs cannot be understood without these semantic elements, which are also far more complex than those contained in nouns. This makes verbs more difficult to understand than nouns. In addition, many studies have also proved that verbs are more difficult to learn than nouns ever since children’s language acquisition (e.g., Gentner, 1982; Gentner and Boroditsky, 2001). Even in languages like Korean, in which verbs often appear at sentence-final positions, children’s acquisition of verbs tends to be later than their acquisition of nouns (Choi and Bowerman, 1991). Adults also find it more difficult to clearly demarcate a verb’s sense than a noun’s. As shown in Gillette et al. (1999), when asked to watch a video in which an artificial verb or noun was said with a referent action to it, and then to specify which verb or noun it was, unlike the rather high accuracy of guessing a noun, adult participants seldom specified a verb’s meaning unless syntactic information was given. Furthermore, research on the human mirror neuron system also reported that: processing verbs tends to recruit not only Broca’s area but also motor cortex for virtual activation, while processing nouns does not need similar mechanisms (Gallese and Lakoff, 2005; Boulenger et al., 2006; Desai et al., 2010, 2011). All these serve as supportive evidence that verbs are far more complicated to learn and understand than nouns. Findings in our study, which excluded syntactic clues, also suggest that the more complicated semantic elements in verbs are essential for the distinct neural substrates between noun and verb processing.

### Noun-Verb-Ambiguous-Words

In our study, significantly different (in amplitude) ERP components were consistently discovered between nouns and noun-verb-ambiguous-words, but not between verbs and noun-verb-ambiguous-words in semantically correct sentences. A possible reason for this is that participants tended to interpret the noun-verb-ambiguous-words as verbs. In general, when they processed the semantically correct sentences, the bottom-up procedure was employed at the beginning to understand each single word’s meaning and its semantic relationship with other words, then, a complete sentence meaning was acquired, and finally, in a top–down manner, the target word’s part-of-speech was specified by analyzing its grammatical function in the sentence. By contrast, if the target word served more than one grammatical function without any morphological changes, and the sentence or background information could not provide more clues, this word would render the whole sentence to be semantically ambiguous, e.g., 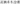 (“This bike has no lockers”/“This bike has not been locked”). People having linguistics training may automatically and immediately notice such ambiguity, but no participants in our study had such disciplined training. In addition, the reason why a word can be a noun-verb ambiguous one is that the verbal and nominal semantics inherent in it have a close rapport between each other, such as 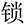 (“locker”/“to lock”).^2^ When asked to make judgment within 2000 ms, participants might not have enough time to notice the hidden ambiguity in those sentences having noun-verb-ambiguous-words. According to the characteristics of human attention, moving objects or dynamic properties are more salient and attractive than static objects or static properties (Franconeri and Simons, 2003; Turatto et al., 2007). Therefore, our participants tended to identify and abstract more of the verbal semantics of noun-verb-ambiguous-words than the nominal semantics, thus prompting them to interpret noun-verb-ambiguous-words more likely as verbs. Saliency difference of semantic components not only affects comprehension of ambiguous words as in our study, but also induces bias in ordering adjectives describing different semantic properties (e.g., color, shape, or texture), as in Gong et al. (2016), which paves the way for simple syntax in language.

## Conclusion

Based on carefully designed Chinese sentences in the configuration of NP + 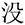 + noun/verb/noun-verb-ambiguous-word, which excludes syntactic priming on the target word’s part-of-speech, we conducted an experimental study demonstrating that processing nouns and verbs consistently evoke different ERP components at different stages of sentence comprehension in native speakers of Chinese. This indicates that semantic factors are essential for the separate neural mechanisms between noun and verb processing. We also reported similar neural differences between noun-verb-ambiguous-words and nouns, suggesting that the verbal semantics of these ambiguous words is more salient than their nominal semantics in the dearth of explicit clues for their part-of-speech, thus inducing participants to interpret them as verbs. This also supports the essential role of semantic factors in the separate substrates between noun and verb processing.

## Data Availability

All datasets generated for this study are included in the manuscript and/or the Supplementary Files.

## Ethics Statement

This study was carried out in accordance with the recommendations of College Research Ethics Committee of Zhejiang University with written informed consent from all subjects. All subjects gave written informed consent in accordance with the Declaration of Helsinki. The protocol was approved by the College Research Ethics Committee.

## Author Contributions

JF and TG designed the research. JF conducted the research. JF, TG, and LS analyzed the results. JF, TG, and YW wrote the manuscript.

## Conflict of Interest Statement

The authors declare that the research was conducted in the absence of any commercial or financial relationships that could be construed as a potential conflict of interest.

## Footnotes

1 www.cncorpus.org

2 There are some common words that contain both verbal and nominal meanings, but no inner relationship exists between the two aspects of meanings. These words are classified as polysemy, such as  in  and  in . Although the two  have not only the same glyph structure but also the same pronunciation, this character is not a noun-verb-ambiguous-word. Because the former  means “flowers” but the latter one means “to spend (money),” there hardly exists any semantic relationship between the two meanings.
