# Supplementary material for: No Morphological Markers, No Problem: ERP Study Reveals Semantic Contribution to Distinct Neural Substrates Between Noun and Verb Processing in Online Sentence Comprehension
Source: Front Neurosci. 2019 Sep 10;13:957. doi: 10.3389/fnins.2019.00957 (PMC6746947; doi:10.3389/fnins.2019.00957)
Supplement: Supplementary file 1 [file Data_Sheet_1.docx]

**No Morphological Markers, No Problem:**

**ERP Study Reveals Semantic Contribution to Distinct Neural Substrates between Noun and Verb Processing in Online Sentence Comprehension**

**Supplementary Materials**

**Table S1.** All the experimental materials divided into 6 conditions, each containing 32 sentences.

| **Condition 1-5** | | | | | |
| --- | --- | --- | --- | --- | --- |
| Condition | NP | Negative word | Target word | Target category | Correctness |
| 1 | 这台相机 | 没 | 包 | Ambiguous | Correct |
| 1 | 这个茶杯 | 没 | 盖 | Ambiguous | Correct |
| 1 | 这辆单车 | 没 | 锁 | Ambiguous | Correct |
| 1 | 这个孩子 | 没 | 尿 | Ambiguous | Correct |
| 1 | 这些人 | 没 | 联系 | Ambiguous | Correct |
| 1 | 这本书 | 没 | 编辑 | Ambiguous | Correct |
| 1 | 这张抽屉 | 没 | 钉 | Ambiguous | Correct |
| 1 | 这件衣服 | 没 | 扣 | Ambiguous | Correct |
| 1 | 这家公司 | 没 | 服务 | Ambiguous | Correct |
| 1 | 这个青年 | 没 | 工作 | Ambiguous | Correct |
| 1 | 这个现象 | 没 | 解释 | Ambiguous | Correct |
| 1 | 这个经理 | 没 | 投资 | Ambiguous | Correct |
| 1 | 这个学生 | 没 | 报告 | Ambiguous | Correct |
| 1 | 这位画家 | 没 | 素描 | Ambiguous | Correct |
| 1 | 这种语言 | 没 | 翻译 | Ambiguous | Correct |
| 1 | 这个社区 | 没 | 代表 | Ambiguous | Correct |
| 1 | 这个女生 | 没 | 选择 | Ambiguous | Correct |
| 1 | 这位老人 | 没 | 安排 | Ambiguous | Correct |
| 1 | 这个句子 | 没 | 标注 | Ambiguous | Correct |
| 1 | 这段时间 | 没 | 规划 | Ambiguous | Correct |
| 1 | 这个电源 | 没 | 保护 | Ambiguous | Correct |
| 1 | 这个大队 | 没 | 装备 | Ambiguous | Correct |
| 1 | 这篇文章 | 没 | 评论 | Ambiguous | Correct |
| 1 | 这个客户 | 没 | 存款 | Ambiguous | Correct |
| 1 | 这次会议 | 没 | 记录 | Ambiguous | Correct |
| 1 | 这个农民 | 没 | 耕地 | Ambiguous | Correct |
| 1 | 这批货物 | 没 | 包装 | Ambiguous | Correct |
| 1 | 这种产品 | 没 | 代理 | Ambiguous | Correct |
| 1 | 这个楼层 | 没 | 监控 | Ambiguous | Correct |
| 1 | 这间屋子 | 没 | 装饰 | Ambiguous | Correct |
| 1 | 这个网站 | 没 | 链接 | Ambiguous | Correct |
| 1 | 这部作品 | 没 | 盗版 | Ambiguous | Correct |
| 2 | 这只兔子 | 没 | 跳 | Verb | Correct |
| 2 | 这瓶可乐 | 没 | 开 | Verb | Correct |
| 2 | 这位客人 | 没 | 走 | Verb | Correct |
| 2 | 这部电影 | 没 | 播 | Verb | Correct |
| 2 | 这封信件 | 没 | 收到 | Verb | Correct |
| 2 | 这种情况 | 没 | 考虑 | Verb | Correct |
| 2 | 这只苹果 | 没 | 洗 | Verb | Correct |
| 2 | 这群学生 | 没 | 来 | Verb | Correct |
| 2 | 这项方案 | 没 | 实行 | Verb | Correct |
| 2 | 这种想法 | 没 | 膨胀 | Verb | Correct |
| 2 | 这面旗帜 | 没 | 悬挂 | Verb | Correct |
| 2 | 这只口哨 | 没 | 吹响 | Verb | Correct |
| 2 | 这个工人 | 没 | 努力 | Verb | Correct |
| 2 | 这只鹦鹉 | 没 | 说话 | Verb | Correct |
| 2 | 这次预算 | 没 | 增加 | Verb | Correct |
| 2 | 这批物资 | 没 | 损耗 | Verb | Correct |
| 2 | 这份文件 | 没 | 处理 | Verb | Correct |
| 2 | 这条新闻 | 没 | 轰动 | Verb | Correct |
| 2 | 这列火车 | 没 | 到达 | Verb | Correct |
| 2 | 这瓶酒精 | 没 | 蒸发 | Verb | Correct |
| 2 | 这个电话 | 没 | 接听 | Verb | Correct |
| 2 | 这颗炸弹 | 没 | 爆炸 | Verb | Correct |
| 2 | 这本小说 | 没 | 阅读 | Verb | Correct |
| 2 | 这对男女 | 没 | 牵手 | Verb | Correct |
| 2 | 这场疾病 | 没 | 恢复 | Verb | Correct |
| 2 | 这次日食 | 没 | 观测 | Verb | Correct |
| 2 | 这项规定 | 没 | 执行 | Verb | Correct |
| 2 | 这位犯人 | 没 | 逃脱 | Verb | Correct |
| 2 | 这张饭卡 | 没 | 挂失 | Verb | Correct |
| 2 | 这份才华 | 没 | 埋没 | Verb | Correct |
| 2 | 这台电脑 | 没 | 运行 | Verb | Correct |
| 2 | 这把椅子 | 没 | 组装 | Verb | Correct |
| 3 | 这位猎人 | 没 | 狗 | Noun | Correct |
| 3 | 这盒电池 | 没 | 电 | Noun | Correct |
| 3 | 这个部落 | 没 | 灯 | Noun | Correct |
| 3 | 这次画展 | 没 | 票 | Noun | Correct |
| 3 | 这条街道 | 没 | 树叶 | Noun | Correct |
| 3 | 这名学生 | 没 | 作品 | Noun | Correct |
| 3 | 这批西瓜 | 没 | 籽 | Noun | Correct |
| 3 | 这座城市 | 没 | 风 | Noun | Correct |
| 3 | 这位作家 | 没 | 稿费 | Noun | Correct |
| 3 | 这套家具 | 没 | 餐桌 | Noun | Correct |
| 3 | 这个部门 | 没 | 主任 | Noun | Correct |
| 3 | 这根电线 | 没 | 胶带 | Noun | Correct |
| 3 | 这名球员 | 没 | 技术 | Noun | Correct |
| 3 | 这名将军 | 没 | 士兵 | Noun | Correct |
| 3 | 这位员工 | 没 | 能力 | Noun | Correct |
| 3 | 这双皮鞋 | 没 | 鞋带 | Noun | Correct |
| 3 | 这个村庄 | 没 | 企业 | Noun | Correct |
| 3 | 这片土地 | 没 | 淡水 | Noun | Correct |
| 3 | 这对夫妻 | 没 | 孩子 | Noun | Correct |
| 3 | 这位护士 | 没 | 手套 | Noun | Correct |
| 3 | 这家餐厅 | 没 | 音乐 | Noun | Correct |
| 3 | 这首古诗 | 没 | 标题 | Noun | Correct |
| 3 | 这种语言 | 没 | 文字 | Noun | Correct |
| 3 | 这种材料 | 没 | 香味 | Noun | Correct |
| 3 | 这个学校 | 没 | 乐队 | Noun | Correct |
| 3 | 这头母象 | 没 | 象牙 | Noun | Correct |
| 3 | 这场比赛 | 没 | 裁判 | Noun | Correct |
| 3 | 这名记者 | 没 | 稿件 | Noun | Correct |
| 3 | 这副耳环 | 没 | 钻石 | Noun | Correct |
| 3 | 这只蜜蜂 | 没 | 翅膀 | Noun | Correct |
| 3 | 这个季节 | 没 | 大风 | Noun | Correct |
| 3 | 这家餐馆 | 没 | 牛肉 | Noun | Correct |
| 4 | 这名罪犯 | 没 | 犯罪 | Verb | Improbable |
| 4 | 这个故事 | 没 | 煮 | Verb | Improbable |
| 4 | 这袋苹果 | 没 | 笑 | Verb | Improbable |
| 4 | 这场沙漠 | 没 | 打 | Verb | Improbable |
| 4 | 这块表 | 没 | 奔跑 | Verb | Improbable |
| 4 | 这只碗 | 没 | 咳嗽 | Verb | Improbable |
| 4 | 这块朽木 | 没 | 腐烂 | Verb | Improbable |
| 4 | 这团火焰 | 没 | 燃烧 | Verb | Improbable |
| 4 | 这群孩子 | 没 | 发生 | Verb | Improbable |
| 4 | 这本日历 | 没 | 跳跃 | Verb | Improbable |
| 4 | 这种习惯 | 没 | 爬山 | Verb | Improbable |
| 4 | 这台电脑 | 没 | 喝水 | Verb | Improbable |
| 4 | 这个意识 | 没 | 打印 | Verb | Improbable |
| 4 | 这个计划 | 没 | 粘贴 | Verb | Improbable |
| 4 | 这种意义 | 没 | 暴动 | Verb | Improbable |
| 4 | 这种麻烦 | 没 | 旋转 | Verb | Improbable |
| 4 | 这把樱桃 | 没 | 努力 | Verb | Improbable |
| 4 | 这张纸巾 | 没 | 建立 | Verb | Improbable |
| 4 | 这首音乐 | 没 | 破产 | Verb | Improbable |
| 4 | 这套工具 | 没 | 呼吸 | Verb | Improbable |
| 4 | 这笔生意 | 没 | 跑步 | Verb | Improbable |
| 4 | 这些材料 | 没 | 免疫 | Verb | Improbable |
| 4 | 这门功课 | 没 | 起飞 | Verb | Improbable |
| 4 | 这滴雨水 | 没 | 打架 | Verb | Improbable |
| 4 | 这次机会 | 没 | 摔倒 | Verb | Improbable |
| 4 | 这块玻璃 | 没 | 思考 | Verb | Improbable |
| 4 | 这枚导弹 | 没 | 洗脸 | Verb | Improbable |
| 4 | 这个数字 | 没 | 冰冻 | Verb | Improbable |
| 4 | 这桶冰块 | 没 | 喝酒 | Verb | Improbable |
| 4 | 这份资料 | 没 | 充电 | Verb | Improbable |
| 4 | 这根铅笔 | 没 | 怒放 | Verb | Improbable |
| 4 | 这块芯片 | 没 | 睡觉 | Verb | Improbable |
| 5 | 这片森林 | 没 | 树木 | Noun | Improbable |
| 5 | 这名歹徒 | 没 | 恶意 | Noun | Improbable |
| 5 | 这块手表 | 没 | 表盘 | Noun | Improbable |
| 5 | 这幢大厦 | 没 | 地基 | Noun | Improbable |
| 5 | 这位哲人 | 没 | 思想 | Noun | Improbable |
| 5 | 这个猛男 | 没 | 肌肉 | Noun | Improbable |
| 5 | 这条彩虹 | 没 | 颜色 | Noun | Improbable |
| 5 | 这只箱子 | 没 | 边框 | Noun | Improbable |
| 5 | 这本词典 | 没 | 词汇 | Noun | Improbable |
| 5 | 这枚电池 | 没 | 负极 | Noun | Improbable |
| 5 | 这场大雨 | 没 | 水分 | Noun | Improbable |
| 5 | 这辆汽车 | 没 | 原子 | Noun | Improbable |
| 5 | 这场大火 | 没 | 温度 | Noun | Improbable |
| 5 | 这个天体 | 没 | 轨道 | Noun | Improbable |
| 5 | 这碗米饭 | 没 | 水稻 | Noun | Improbable |
| 5 | 这个图形 | 没 | 形状 | Noun | Improbable |
| 5 | 这位天才 | 没 | 智商 | Noun | Improbable |
| 5 | 这个美女 | 没 | 容貌 | Noun | Improbable |
| 5 | 这剂猛药 | 没 | 药效 | Noun | Improbable |
| 5 | 这个胖子 | 没 | 脂肪 | Noun | Improbable |
| 5 | 这个圆环 | 没 | 圆心 | Noun | Improbable |
| 5 | 这次成功 | 没 | 原因 | Noun | Improbable |
| 5 | 这座大山 | 没 | 高度 | Noun | Improbable |
| 5 | 这块巨石 | 没 | 重量 | Noun | Improbable |
| 5 | 这种天气 | 没 | 气候 | Noun | Improbable |
| 5 | 这段历史 | 没 | 故事 | Noun | Improbable |
| 5 | 这枚硬币 | 没 | 反面 | Noun | Improbable |
| 5 | 这种振动 | 没 | 频率 | Noun | Improbable |
| 5 | 这个矩形 | 没 | 直角 | Noun | Improbable |
| 5 | 这条裤子 | 没 | 裤筒 | Noun | Improbable |
| 5 | 这颗子弹 | 没 | 弹壳 | Noun | Improbable |
| 5 | 这勺蜂蜜 | 没 | 糖分 | Noun | Improbable |

| Condition 6 | | |
| --- | --- | --- |
| NP | VP | Correctness |
| 这只蜜蜂 | 吃蟒蛇 | Improbable |
| 这叠发票 | 爱说话 | Improbable |
| 这筐网球 | 抽香烟 | Improbable |
| 这车面包 | 打太极 | Improbable |
| 这头鲸鱼 | 登珠峰 | Improbable |
| 这把菜刀 | 跳街舞 | Improbable |
| 这本字典 | 上台阶 | Improbable |
| 这幅油画 | 打篮球 | Improbable |
| 这面墙壁 | 闹情绪 | Improbable |
| 这本字典 | 砍大树 | Improbable |
| 这块地板 | 描眉毛 | Improbable |
| 这捆木柴 | 打电话 | Improbable |
| 这只蚯蚓 | 练拳击 | Improbable |
| 这片池塘 | 戴眼镜 | Improbable |
| 这颗卫星 | 吹空调 | Improbable |
| 这张饭卡 | 看电视 | Improbable |
| 这块钢板 | 吹牛皮 | Improbable |
| 这截毛线 | 铺地板 | Improbable |
| 这朵云彩 | 盖高楼 | Improbable |
| 这口大锅 | 写作业 | Improbable |
| 这个信封 | 说大话 | Improbable |
| 这台电视 | 炸薯条 | Improbable |
| 这根钢筋 | 拉肚子 | Improbable |
| 这台风扇 | 喝牛奶 | Improbable |
| 这片真空 | 做体检 | Improbable |
| 这桶冰块 | 倒垃圾 | Improbable |
| 这只蚂蚁 | 穿皮鞋 | Improbable |
| 这位光棍 | 秀恩爱 | Improbable |
| 这盏台灯 | 跑得快 | Improbable |
| 这只螃蟹 | 梳头发 | Improbable |
| 这个牙刷 | 种玉米 | Improbable |
| 这截木头 | 吃面条 | Improbable |

**Table S2.** Static results for ACC. Top: Two-way repeated measures ANOVA; Bottom: One-way repeated measures ANOVA.

|  | ***F*** (1, 29) | ***p*** | ***η*^2^** | **Separate pair-wised *t* test** | | | | | |
| --- | --- | --- | --- | --- | --- | --- | --- | --- | --- |
| **Lexical category** (verbs / nouns) | 19.356 | < .001 | .4 | verbs vs. nouns in semantically correct sentences | | | verbs vs. nouns in semantically improbable sentences | | |
| **Sentence semantic correctness** (correct / improbable) | 2.553 | .121 | .081 | ***t*** | ***df*** | ***p*** | ***t*** | ***df*** | ***p*** |
| **Lexical category × Sentence semantic correctness** | 2.597 | .119 | .082 | 3.179 | 31 | .003 | 2.918 | 31 | .007 |

|  | ***F*** (1, 29) | ***p*** | ***η*^2^** | **Separate pair-wised *t* test** | | | | | | | | |
| --- | --- | --- | --- | --- | --- | --- | --- | --- | --- | --- | --- | --- |
| **Lexical category** (noun-verb-ambiguous-words / verbs / nouns) | 7.96 | .003 | .215 | noun-verb-ambiguous-words vs. verbs | | | noun-verb-ambiguous-words vs. nouns | | | verbs vs. nouns | | |
|  |  |  |  | ***t*** | ***df*** | ***p*** | ***t*** | ***df*** | ***p*** | ***t*** | ***df*** | ***p*** |
|  |  |  |  | -.325 | 31 | .747 | 2.663 | 31 | .012 | 3.179 | 31 | .003 |

**Table S3.** Static results for RT. Top: Two-way repeated measures ANOVA; Bottom: One-way repeated measures ANOVA.

|  | ***F*** (1, 29) | ***p*** | ***η*^2^** |
| --- | --- | --- | --- |
| **Lexical category** (verbs / nouns) | 2.117 | .156 | .068 |
| **Sentence semantic correctness** (correct / improbable) | 2.031 | .165 | .065 |
| **Lexical category × Sentence semantic correctness** | .392 | .536 | .013 |

|  | ***F*** (1, 29) | ***p*** | ***η*^2^** |
| --- | --- | --- | --- |
| **Lexical category** (noun-verb-ambiguous-words / verbs / nouns) | 2.283 | .132 | .073 |

**Table S4.** Static results for P200. Top: Two-way repeated measures ANOVA; Bottom: One-way repeated measures ANOVA.

|  | ***F*** (1, 29) | ***p*** | ***η*^2^** | **Separate pair-wised *t* test** | | | | | |
| --- | --- | --- | --- | --- | --- | --- | --- | --- | --- |
| **Lexical category** (verbs / nouns) | 67.159 | < .001 | .698 | verbs vs. nouns in semantically correct sentences | | | verbs vs. nouns in semantically improbable sentences | | |
| **Sentence semantic correctness** (correct / improbable) | 1.436 | .24 | .047 | ***t*** | ***df*** | ***p*** | ***t*** | ***df*** | ***p*** |
| **Lexical category × Sentence semantic correctness** | .213 | .648 | .007 | 4.768 | 31 | < .001 | 6.53 | 31 | < .001 |

|  | ***F*** (1, 29) | ***p*** | ***η*^2^** | **Separate pair-wised *t* test** | | | | | | | | |
| --- | --- | --- | --- | --- | --- | --- | --- | --- | --- | --- | --- | --- |
| **Lexical category** (noun-verb-ambiguous-words / verbs / nouns) | 14.063 | < .001 | .327 | noun-verb-ambiguous-words vs. verbs | | | noun-verb-ambiguous-words vs. nouns | | | verbs vs. nouns | | |
|  |  |  |  | ***t*** | ***df*** | ***p*** | ***t*** | ***df*** | ***p*** | ***t*** | ***df*** | ***p*** |
|  |  |  |  | -.229 | 31 | .820 | 5.005 | 31 | < .001 | 4.768 | 31 | < .001 |

**Table S5.** Static results for N400. Top: Two-way repeated measures ANOVA; Bottom: One-way repeated measures ANOVA.

|  | ***F*** (1, 29) | ***p*** | ***η*^2^** | **Separate pair-wised *t* test** | | | | | | | | | | | |
| --- | --- | --- | --- | --- | --- | --- | --- | --- | --- | --- | --- | --- | --- | --- | --- |
| **Lexical category** (verbs / nouns) | 23.445 | < .001 | .447 | verbs vs. nouns in semantically correct sentences | | | verbs vs. nouns in semantically improbable sentences | | | verbs in semantically correct vs. improbable sentences | | | nouns in semantically correct vs. improbable sentences | | |
| **Sentence semantic correctness** (correct / improbable) | 185.976 | < .001 | .865 | ***t*** | ***df*** | ***p*** | ***t*** | ***df*** | ***p*** | ***T*** | ***df*** | ***p*** | ***t*** | ***df*** | ***p*** |
| **Lexical category × Sentence semantic correctness** | 5.556 | .025 | .161 | -4.852 | 31 | < .001 | -.626 | 31 | .536 | 8.211 | 31 | < .001 | 14.897 | 31 | < .001 |

|  | ***F*** (1, 29) | ***p*** | ***η*^2^** | **Separate pair-wised *t* test** | | | | | | | | |
| --- | --- | --- | --- | --- | --- | --- | --- | --- | --- | --- | --- | --- |
| **Lexical category** (noun-verb-ambiguous-words / verbs / nouns) | 13.318 | < .001 | .315 | noun-verb-ambiguous-words vs. verbs | | | noun-verb-ambiguous-words vs. nouns | | | verbs vs. nouns | | |
|  |  |  |  | ***t*** | ***df*** | ***p*** | ***t*** | ***df*** | ***p*** | ***t*** | ***df*** | ***p*** |
|  |  |  |  | -.040 | 31 | .969 | -4.632 | 31 | < .001 | -4.852 | 31 | < .001 |

**Table S6.** Static results for P600. Top: Two-way repeated measures ANOVA; Bottom: One-way repeated measures ANOVA.

|  | ***F*** (1, 29) | ***p*** | ***η*^2^** | **Separate pair-wised *t* test** | | | | | | | | | | | |
| --- | --- | --- | --- | --- | --- | --- | --- | --- | --- | --- | --- | --- | --- | --- | --- |
| **Lexical category** (verbs / nouns) | 9.412 | .005 | .245 | verbs vs. nouns in semantically correct sentences | | | verbs vs. nouns in semantically Improbable sentences | | | verbs in semantically correct vs. Improbable sentences | | | nouns in semantically correct vs. Improbable sentences | | |
| **Sentence semantic correctness** (correct / improbable) | 189.503 | < .001 | .867 | ***t*** | ***df*** | ***p*** | ***t*** | ***df*** | ***p*** | ***t*** | ***df*** | ***p*** | ***t*** | ***df*** | ***p*** |
| **Lexical category × Sentence semantic correctness** | 1.183 | .286 | .039 | 7.491 | 31 | < .001 | -.903 | 31 | .374 | 18.407 | 31 | < .001 | 11.181 | 31 | < .001 |

|  | ***F*** (1, 29) | ***p*** | ***η*^2^** | **Separate pair-wised *t* test** | | | | | | | | |
| --- | --- | --- | --- | --- | --- | --- | --- | --- | --- | --- | --- | --- |
| **Lexical category** (noun-verb-ambiguous-words / verbs / nouns) | 21.016 | < .001 | .42 | noun-verb-ambiguous-words vs. verbs | | | noun-verb-ambiguous-words vs. nouns | | | verbs vs. nouns | | |
|  |  |  |  | ***t*** | ***df*** | ***p*** | ***t*** | ***df*** | ***p*** | ***t*** | ***df*** | ***p*** |
|  |  |  |  | .367 | 31 | .716 | 9.477 | 31 | < .001 | 7.491 | 31 | < .001 |


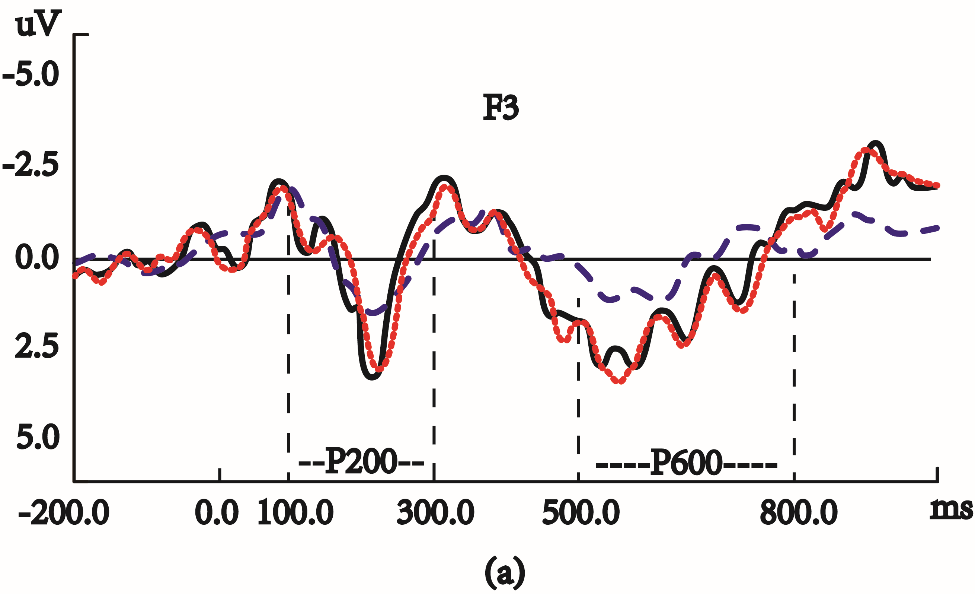

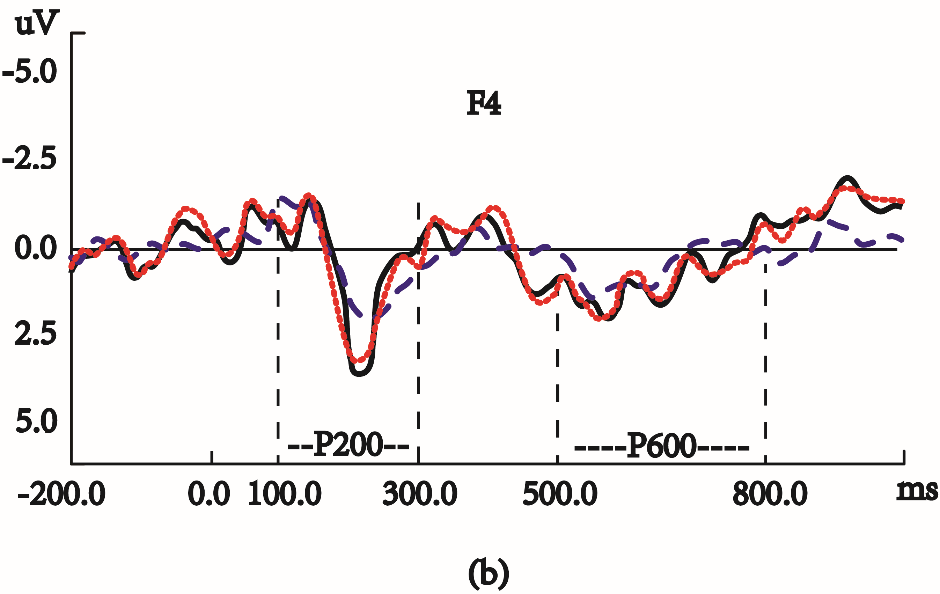


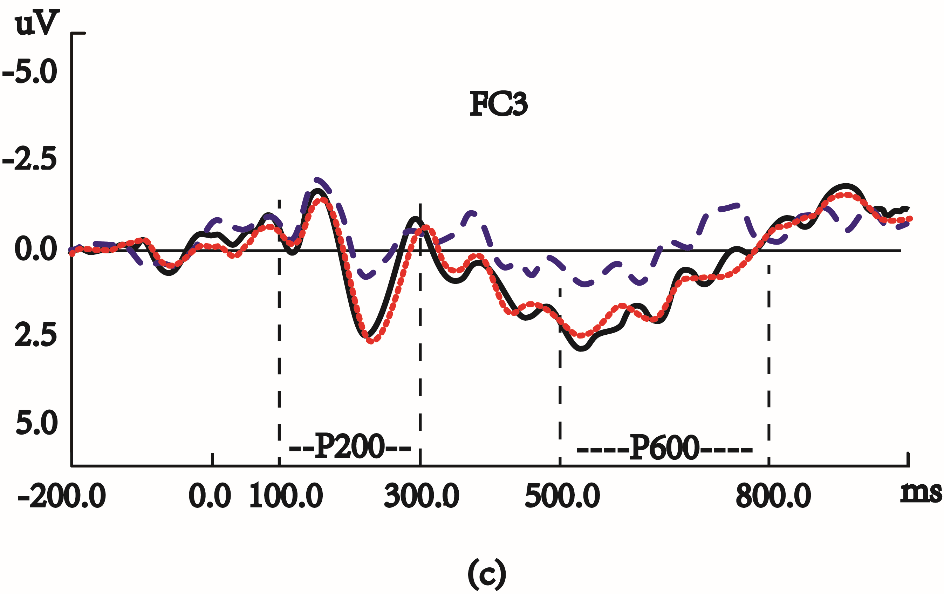

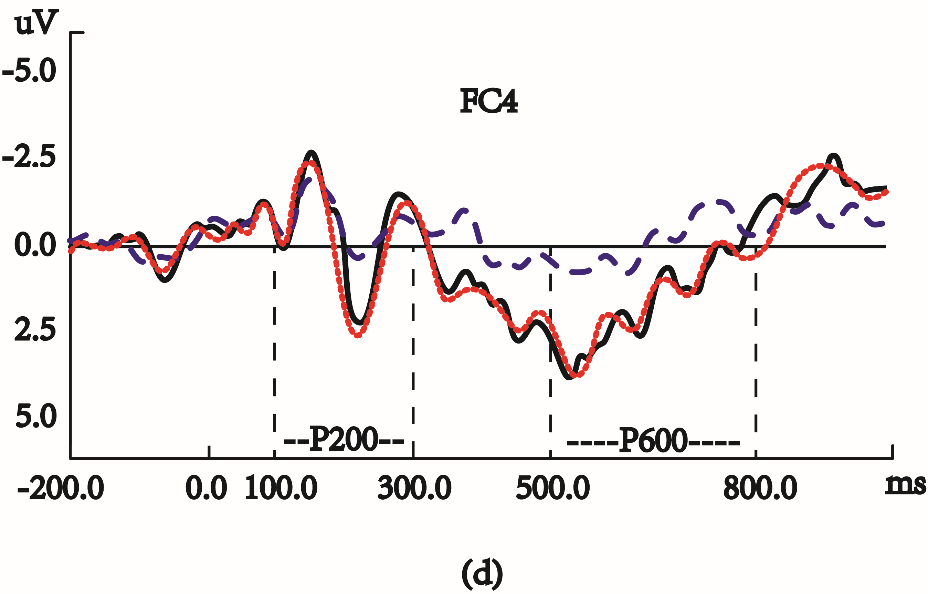


**Fig. S1.** Average waves elicited by the target nouns (blue dashed lines), verbs (black solid lines), and noun-verb-ambiguous-words (red dotted lines) during the P200 (within left two vertical lines) and P600 (within the right two vertical lines) time windows at electrodes F3 (a), F4 (b), FC3 (c) and FC4 (d) in the semantically correct sentences.

**
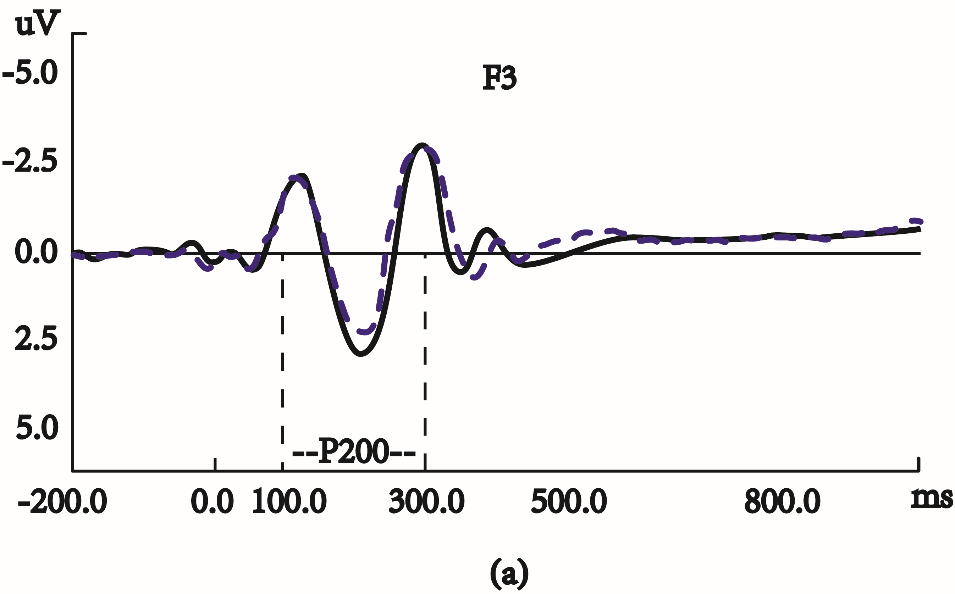

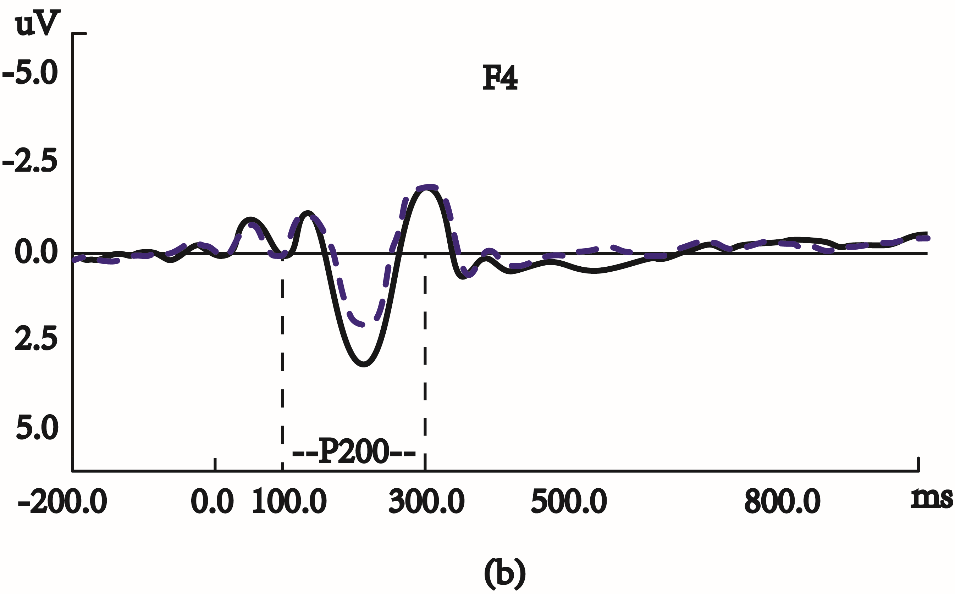
**

**
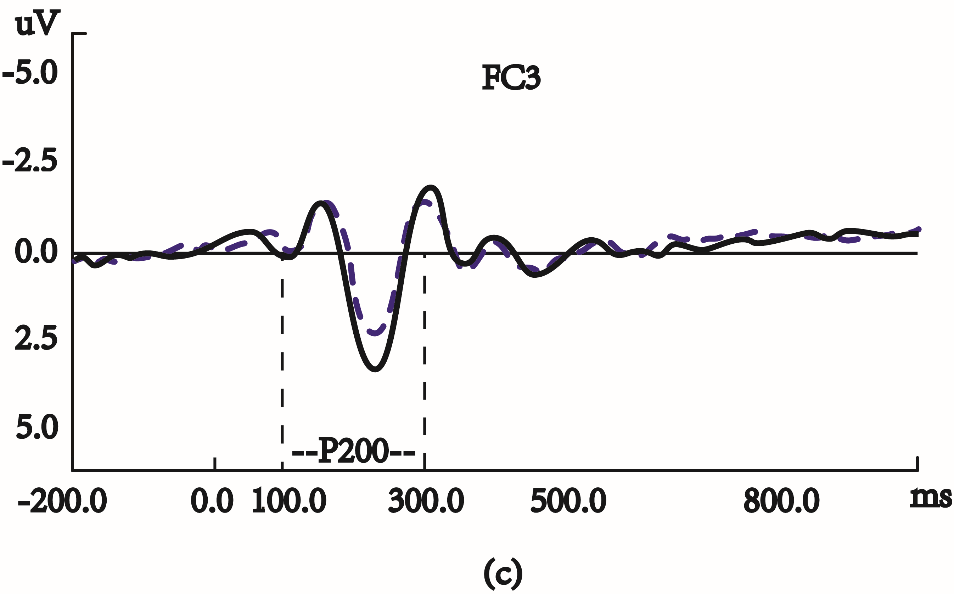

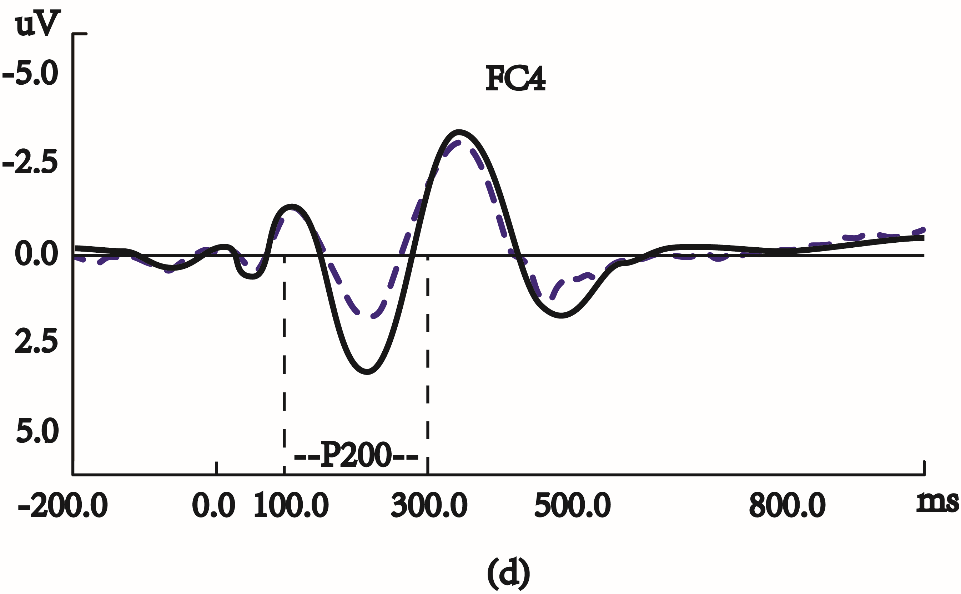
**

**Fig. S2.** Average waves elicited by the target nouns (blue dashed lines) and verbs (black solid lines) during the P200 time windows (within left two vertical lines) at electrodes F3 (a), F4 (b), FC3 (c) and FC4 (d) in the semantically improbable sentences.


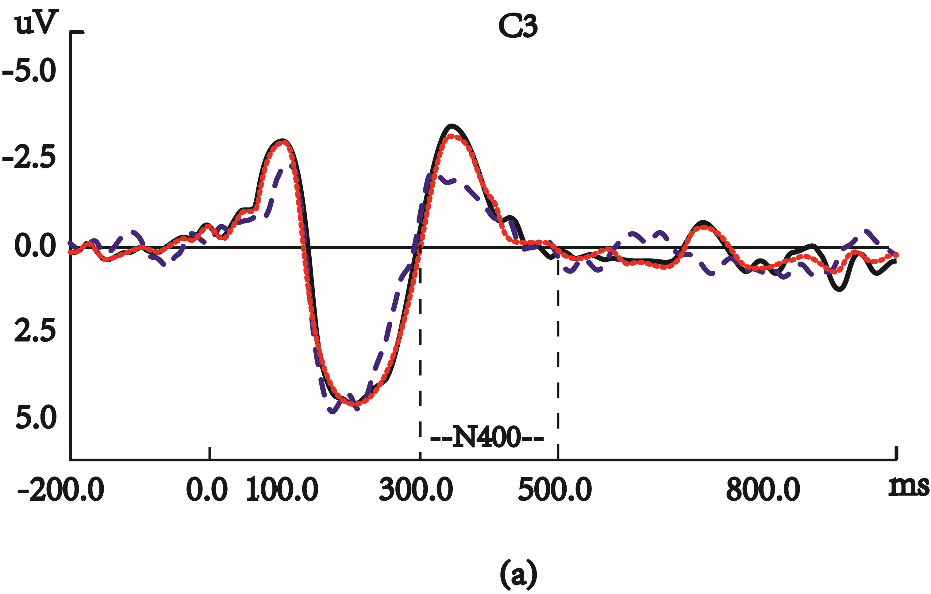

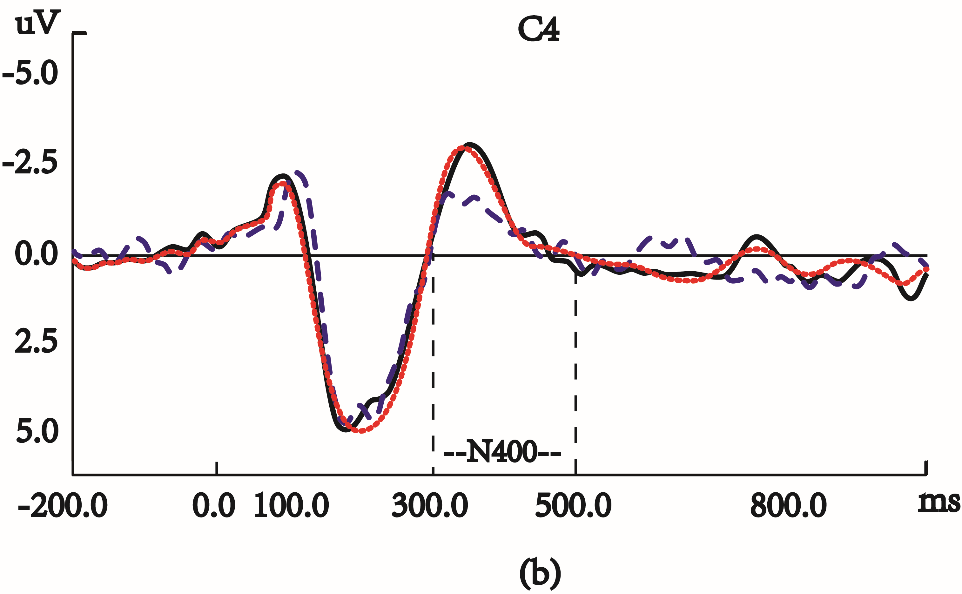


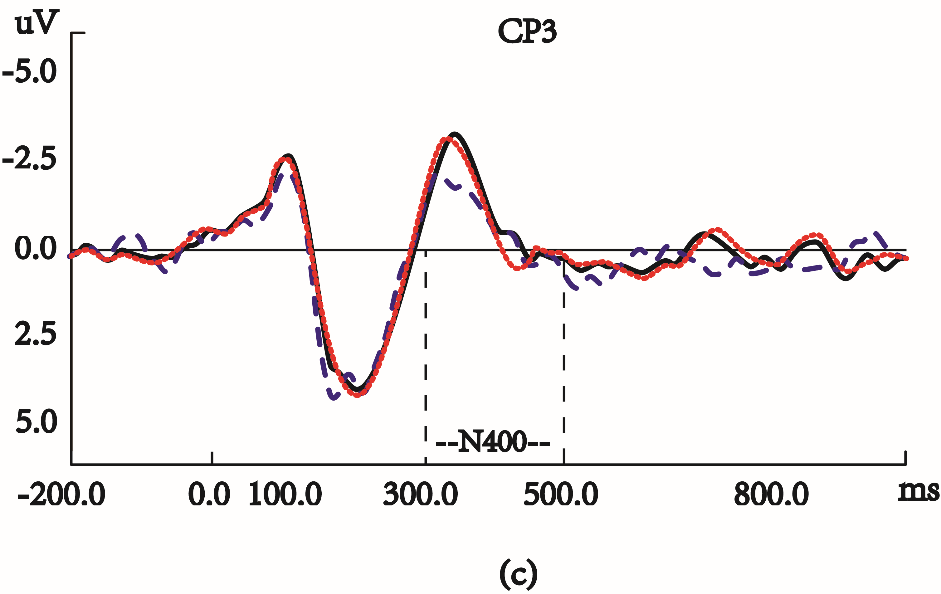

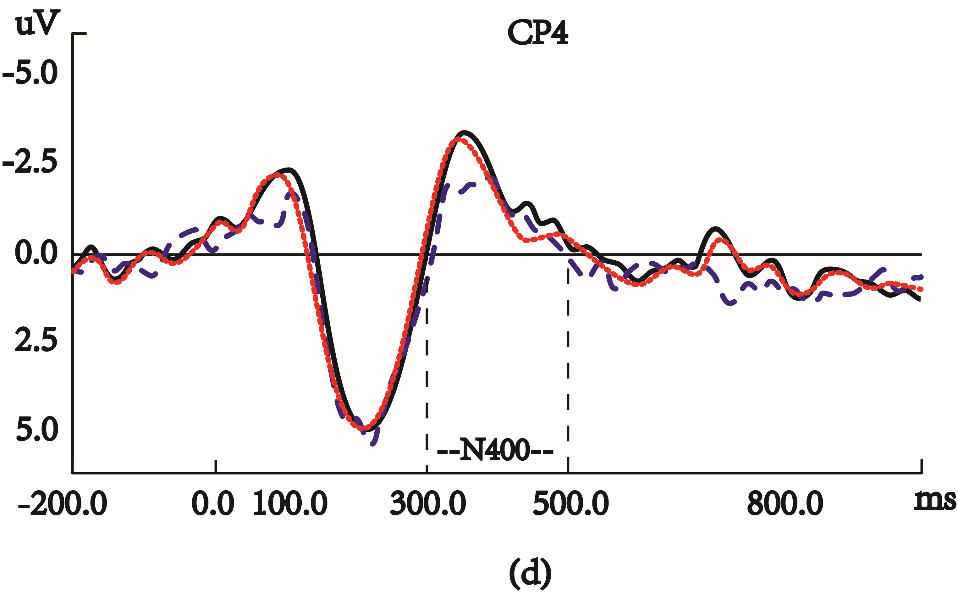


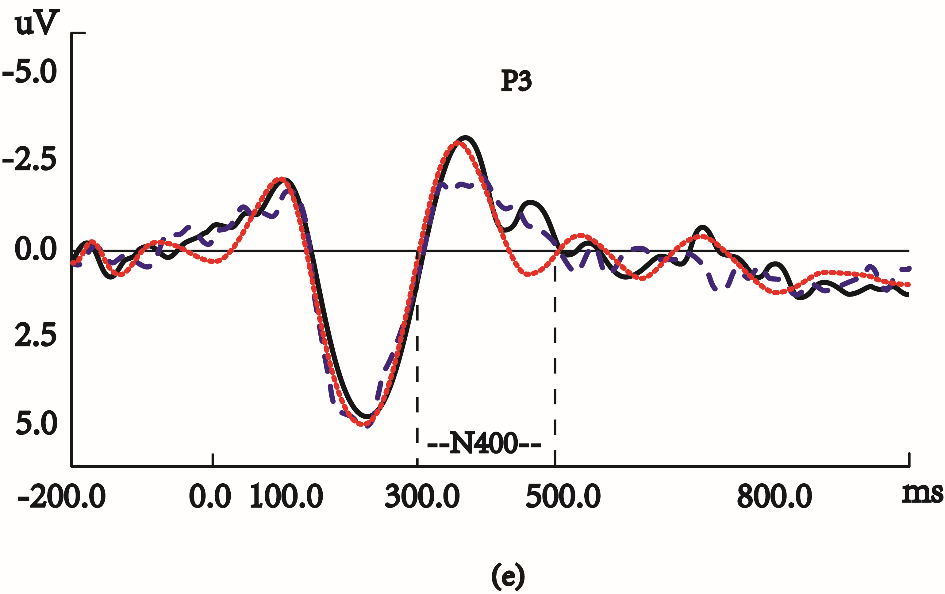

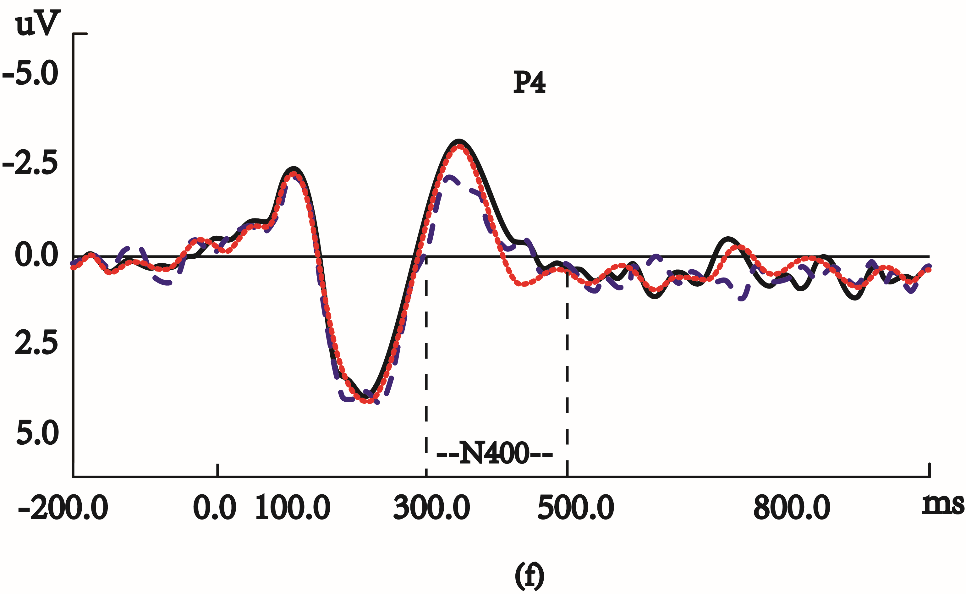


**Fig. S3.** Average waves elicited by the target nouns (blue dashed lines), verbs (black solid lines), and noun-verb-ambiguous-words (red dotted lines) during the N400 time window (within the two vertical lines) at electrodes C3 (a), C4 (b), CP3 (c), CP4 (d), P3 (e) and P4 (f) in the semantically correct sentences.


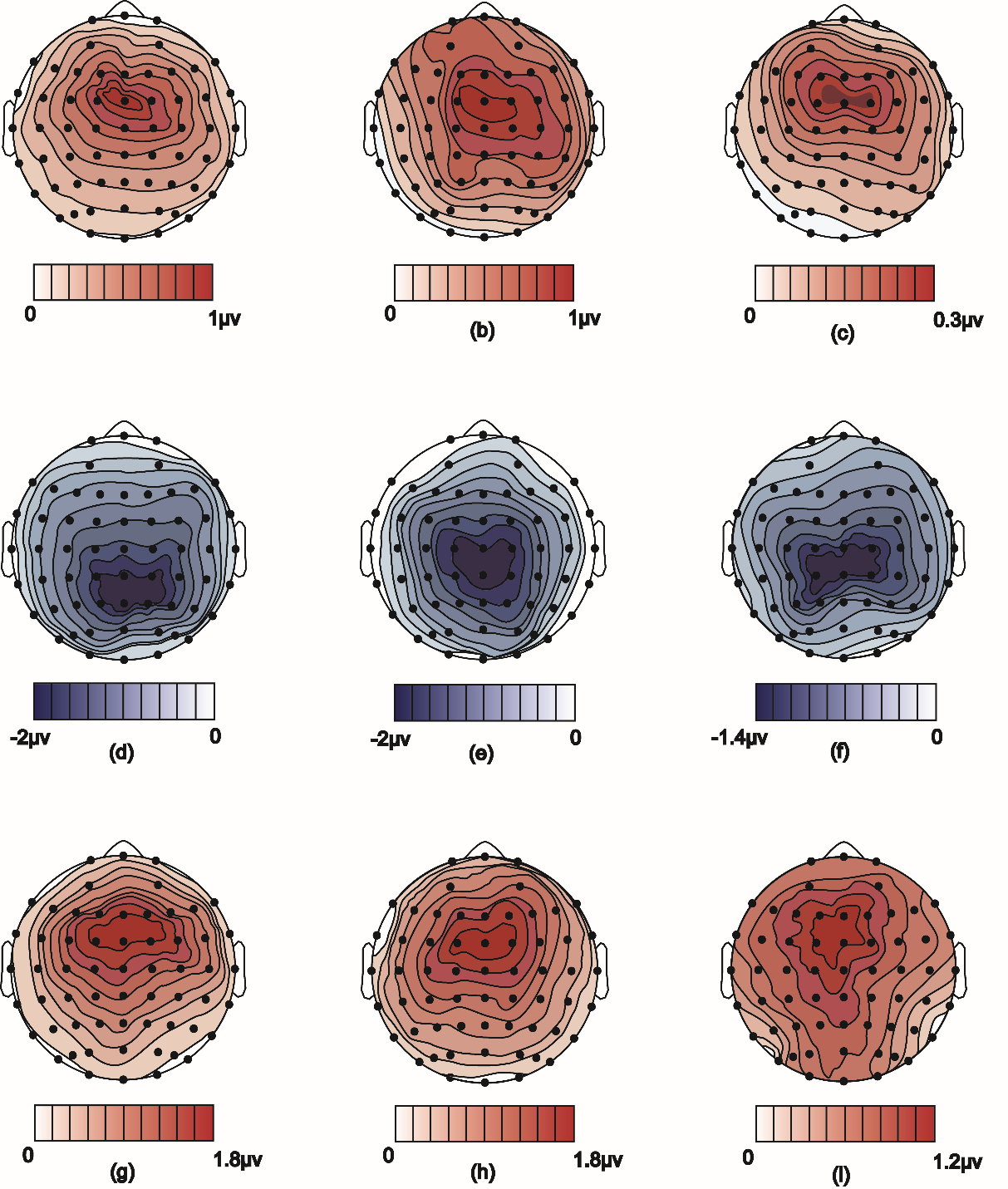


**Fig. S4.** Topographies of the ERP components in the semantically correct sentences. (a)-(c) are the P200 (100-300 ms) of the target noun-verb-ambiguous-words, verbs and nouns, respectively; (d)-(f) are the N400 (300-500 ms) of noun-verb-ambiguous-words, verbs, and nouns, respectively; and (g)-(i) are the P600 (500-800 ms) of noun-verb-ambiguous-words, verbs, and nouns, respectively.


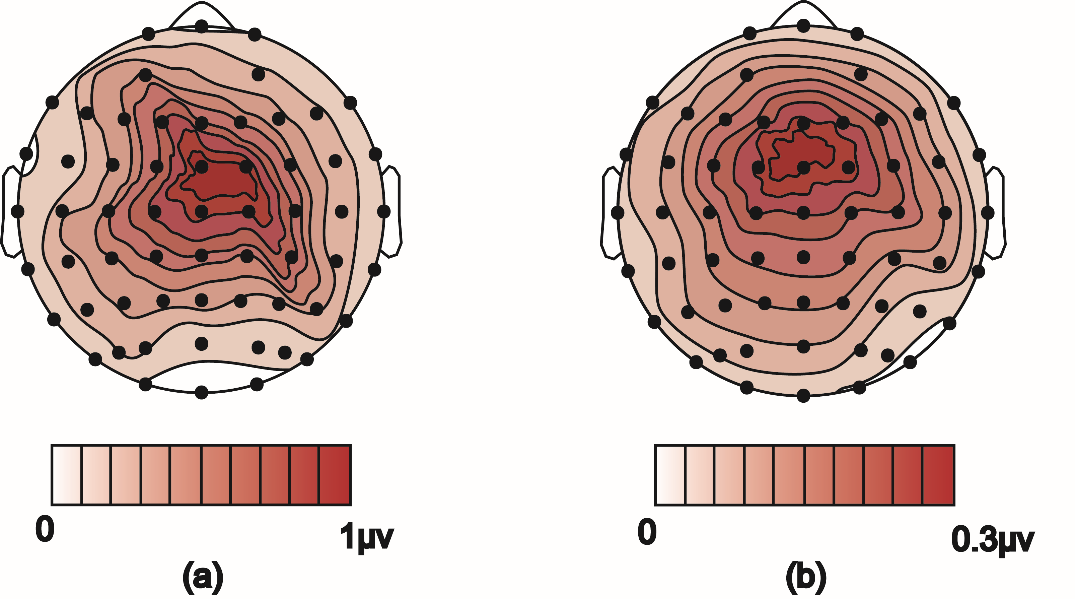


**Fig. S5.** Topographies of P200 (100-300 ms) in the semantically improbable sentences. (a) and (b) are for verbs and nouns, respectively.
